# The #chatsafe Youth Participation Model: Evaluating Participatory Workshops Involving Young People in the Design of Suicide Prevention Initiatives

**DOI:** 10.1111/hex.70885

**Published:** 2026-10-03

**Authors:** L. La Sala, E. R. Carrotte, C. Cooper, M. Lamblin, E. Brown, J. Robinson

**Affiliations:** ^1^ Orygen, The National Centre of Excellence in Youth Mental Health Parkville Victoria Australia; ^2^ Centre for Youth Mental Health, The University of Melbourne Parkville Victoria Australia

**Keywords:** co‐design, evaluation, participatory methods, social media, suicide prevention, young people

## Abstract

**Background:**

#chatsafe is a programme aiming to empower young people to communicate safely on social media about self‐harm and suicide. This study aimed to assess the feasibility, acceptability, safety, and impact associated with attending a #chatsafe participatory workshop, using the #chatsafe Youth Participation Model. The workshops aimed to educate young people on safe communication practices and gather their insights to inform new #chatsafe social media intervention content.

**Methods:**

Quantitative data were collected in a pre‐ and post‐workshop survey using bespoke items measuring perceived feasibility, acceptability, and safety of the workshops, and self‐efficacy in communicating online about suicide. Qualitative data were collected during the workshop (via participant feedback, field notes, audio recordings, and facilitator debriefing notes) and in open‐text responses in the post‐workshop survey.

**Results:**

Nine workshops were held with 107 participants (*age M* = 19.7 years, SD = 2.7 years). Two thirds (64.5%) reported lifetime suicidal thoughts. Eighty‐seven (81.3%) completed both the pre‐ and post‐workshop survey. Feedback was positive, with 94.3% of participants reporting satisfaction with the workshop, 96.5% reported enjoying the workshop, and no adverse events were recorded. There was a significant increase in confidence when communicating online about suicide following participation in a #chatsafe workshop, *z* = −5.99, *p* < 0.001. Workshop insights informed two national Australian #chatsafe Instagram campaigns, that reached 950,000 young people and 818,000 young people respectively.

**Conclusions:**

The #chatsafe workshops were highly feasible, acceptable, safe, and led to young people feeling better equipped to communicate safely online about suicide. To further encourage the involvement of young people in the design of suicide prevention, mental health, and similar initiatives, a #chatsafe Youth Participation Toolkit is publicly available for other researchers and outlines the steps taken to plan, conduct, and translate the outcomes of such workshops.

**Lived Experience or Public Contribution:**

This study summarises the views of 87 young people who attended a #chatsafe participatory workshop and provided feedback. The #chatsafe Youth Participation Toolkit was also shaped by seven workshop participants who attended a subsequent focus group, which focused on what recommendations the researchers should share with others who wish to use these participatory methods in the future.

## 1 Introduction

Suicide remains the leading cause of death among young people in Australia [1]. Despite substantial and sustained national and global investment, rates of suicide and self‐harm amongst young people have not meaningfully shifted [1]. This raises critical questions about the effectiveness and appropriateness of current suicide prevention initiatives. One potential explanation is the limited involvement of end‐users in the design, development, and implementation of current youth suicide prevention approaches [2]. There have been calls for increased youth involvement in the field of suicide prevention [3, 4], including within Australia [5].

Participatory research is ‘a research‐to‐action approach that emphasises direct engagement of local priorities and perspectives’ [6]. Participatory research can generate creative ideas and more impactful interventions [7, 8, 9] and has become increasingly common over the last 15 years [10]. However, people with lived experience of suicidal thoughts and self‑harm are still rarely involved as partners in suicide prevention research [2, 3, 11, 12, 13]. Notably, one systematic review found that out of 99 studies examining suicide prevention interventions targeting young people, none reported participatory research involving young people in the design of the intervention or subsequent research [14].

Ethical and practical concerns have been raised regarding youth participation in suicide research, such as fears that participation may increase distress or suicide risk [15, 16, 17]. However, when conducted ethically and with age‐appropriate safeguards, involving young people in suicide prevention research can be both safe and beneficial, including for young people with lived experience [4, 16, 18, 19]. Studies show that participants report altruism, personal growth and self‑insight, value connecting with peers and researchers, and appreciate having their voices heard [18, 20, 21]. However, there are also process‐related challenges; participatory research is often resource‐intensive and iterative, particularly for co‐design or co‐production activities that may take months or even years [22]. Participatory research may also fail to balance power, and risks tokenistic involvement that can lead to frustration, disempowerment, and hesitation to engage in future youth participation activities [20, 22, 23].

Considerations for youth participatory research in suicide prevention include completion of wellness plans, clear communication and informed consent procedures, safe sharing agreements, flexible participation options, detailed safety protocols, and referral information for mental health and crisis services [17]. Although the values underlying participatory involvement are well documented (e.g [10, 23, 24, 25]), many research papers remain broad and offer limited practical guidance for generalising procedures (e.g [2, 24, 26]). This limits both replicability and the evidence base for many participatory approaches. Few existing studies detail the measures or protocols adopted to support participant safety and wellbeing [2], even though this is an important consideration for participatory suicide prevention research [4]. Furthermore, evaluation of participatory research is generally lacking, especially in the context of youth and/or lived experience participation in suicide prevention research [2, 27].

### 1.1 The #chatsafe Youth Participation Model

The #chatsafe Youth Participation Model (the ‘#chatsafe Model’) aims to meaningfully involve young people aged 15–25 years in creative design activities related to suicide prevention, mental ill‑health and other sensitive topics. This approach was developed in the suicide prevention context as part of the #chatsafe programme [28, 29]. Considering the varied harms and benefits for social media in the context of suicide and self‐harm [30], #chatsafe was created to empower young people with the skills to communicate safely on social media about self‐harm and suicide. #chatsafe comprises a set of evidence‐informed guidelines [28], reaching young people, parents, carers and communities, through social media content that promotes the guidelines' messaging. The social media campaign is used as both a universal suicide prevention intervention, and as a postvention approach, through deliberate deployment of specific content for communities impacted by suicide [31, 32]. To date, two pre‐post evaluations of the #chatsafe social media intervention have found the approach to be safe, acceptable, and effective [31, 33], and results of a randomised controlled trial are being prepared for publication [34].

Young people have been involved in the design of #chatsafe campaigns since inception through participatory workshops [15]. To date, 28 Australian workshops have been delivered with over 300 young people; three international workshops have also occurred (Norway, Italy and Bermuda). Young people have previously found these to be safe and enjoyable [15]. However, since the initial workshop series, #chatsafe guidelines have been updated [29] and the #chatsafe Model has been refined, drawing on best practice guidelines for involving young people with lived experience in suicide research [4], and other participatory approaches [35]. To date, the #chatsafe Model has not been described in detail, nor has it been rigorously evaluated.

This current study had four aims, addressing both the participatory process (aims 1 and 2) and workshop outcomes (3 and 4):

1. To assess the feasibility, acceptability, and safety associated with participating in a #chatsafe workshop, based on the #chatsafe Model.
2. To explore whether participation in the #chatsafe workshop increases a young person's perceived self‐efficacy to communicate safely online about self‐harm or suicide.
3. To design a new social media campaign with young people that reflected information from Edition 2 of the guidelines.
4. To measure the reach and engagement of subsequent #chatsafe social media campaign content directly informed by the workshops.

## 2 Methods

### 2.1 Design

This was an evaluation of a series of workshops conducted using the #chatsafe Model, based on indicators adapted from the Youth Engagement Evaluation Framework [36] (see Appendix 1), using an embedded mixed methods design where quantitative and qualitative data are collected simultaneously [37]. The study was approved by the University of Melbourne Human Research Ethics Committee (2024‐26610‐61292‐10).

### 2.2 Participants

Eligibility criteria to attend a #chatsafe workshop required that participants were: (1) aged 15‐25 years old, (2) living in Australia, (3) able to speak and read English, (4) able to attend the full workshop (~ 6 h), and (5) had not previously participated in a #chatsafe workshop. In total, nine workshops were conducted, targeting either a general youth audience (*k* = 3) or one of four priority groups (*k* = 6): culturally and linguistically diverse young people (*k* = 1), young people with lived experience of self‐harm and/or suicide (*k* = 1), Lesbian, Gay, Bisexual, Transgender and Queer (LGBTQ + ) young people (*k* = 1), and school‐aged adolescents aged 15‐18 years (*k* = 3).

Participants were both campaign co‐designers and evaluation research participants. Workshops were designed for 8–30 participants, with most expected to involve 10–20 participants, aligned with previous research using similar participatory approaches [38]. Recruitment used convenience sampling via organic and paid social media advertising, digital flyers, and emails to existing networks. Partner organisations, such as youth advocacy groups and youth mental health organisations, were invited to co‑host workshops and/or share recruitment information, by posting to their social media accounts, emailing their youth networks, and displaying flyers in communal areas (e.g. waiting rooms). For priority population workshops, the #chatsafe team partnered with population‐specific organisations (including a LGBTQ+ youth organisation and a multicultural youth organisation) and/or adjusted wording and imagery in recruitment materials.

### 2.3 Safety Considerations

A safety strategy was adapted from the original #chatsafe workshop series [15] and aligned with best practice guidelines [4]. All workshops involved ice breaker activities, a facilitated ‘room agreement’ discussion to help establish shared group norms, and cool‐down activities. Together, these activities were designed to build psychological safety, develop rapport, and reduce the likelihood of distress. Participants were provided with information about Australian youth mental health and suicide prevention helplines before, during, and after the workshop.

During registration, participants completed an online wellness plan capturing contact details and optional prompts relating to mental health information and coping strategies [20]. A staff member in a designated wellbeing role used these to assist with monitoring for potential discomfort or distress. Participants were also asked to notify a facilitator if they were feeling distressed or needed a break. A psychologist was on‐call to provide consultation to facilitators or assist with management of any identified safety concerns or risk of harm. Sensory toys and a quiet breakout space were available to all participants.

Finally, the post‑workshop survey included items assessing whether the workshop made participants feel upset, suicidal or want to self‑harm. Agreeing with any of these items triggered a pop‐up message reiterating helpline‐information and advising they could contact the study team if desired, alongside the option to stop the survey. Selecting this option generated an email alert to the team to process their withdrawal. No action was taken unless withdrawal was requested, in which case they were offered the opportunity to speak with a study psychologist over the phone. Participants were shown helpline information at the end of the Time 2 (T2) survey regardless of their responses, and this was also emailed post‐workshop.

### 2.4 Measures

#### 2.4.1 Demographics

At Time 1 (T1, pre‐workshop), participants reported demographic information (age, gender identity, sexuality, language spoken at home, cultural or ethnic group, Aboriginal or Torres Strait Islander identity, employment/student status, and education). Open‑text responses were reviewed and categorised. Lived or living experience of self‑harm or suicide was assessed with yes/no questions about lifetime self‑harm ideation and behaviour, suicidal thoughts and suicide attempts, and about exposure to a suicide death of someone they knew.

#### 2.4.2 Evaluation Items

Survey questions comprised purpose‐designed Likert and optional open text items aligned with the evaluation indicators [36] (see Appendix 1). Acceptability was measured at Time 2 (T2, post‐workshop) through assessment of participants' views on the workshop's relevance, inclusivity, usefulness, helpfulness, and their overall satisfaction and enjoyment. Next, feasibility was measured based on the number of young people recruited through the sampling approach per workshop (including priority population workshops), as well as representation of these demographics across the workshop series altogether. It was also informed by the participants' perceptions of workshop accessibility at T2. Safety was measured through assessing distress, suicidal ideation and self‐harm reported at T2, and the number of adverse events occurring during workshops.

Finally, self‐efficacy was operationalised as self‐perceived confidence, knowledge, and skill when communicating online about self‐harm and suicide. As no validated measure existed at the time of data collection, we developed six items relating to confidence (Cronbach's α = 0.83) on a five‐point Likert Scale from ‘Not at all confident’ (1) to ‘Very confident’ (5). Higher scores indicated a higher degree of confidence. At both T1 and T2, participants were shown the prompt: ‘The following statements will ask you about your confidence levels in communicating safely online about self‐harm and suicide.’ The six items covered the following domains in relation to suicide and self‐harm content: (1) having a conversation, (2) starting a conversation, (3) using safe language, (4) recognising harmful content, (5) responding to content, and (6) reporting content. Participants were also asked at T2 if the workshop ‘led to improved knowledge about safe online communication about self‐harm and suicide’ (rated *Yes/No/Unsure*) and ‘improved [their] ability to communicate safely online about self‐harm and suicide’ (rated *Didn't improve my ability much, Somewhat improved my ability, Improved my ability*, and *Strongly improved my ability*).

### 2.5 Procedure

Procedures were adapted from the initial #chatsafe workshop series [15] and were aligned with the #chatsafe Model (see toolkit for detail – Appendix 3). All registrations and evaluation data were managed through REDCap software platform hosted through the University of Melbourne [39, 40]. Participants completed an eligibility screener and registration form including a plain language statement, consent form, wellness plan, demographic and banking details and T1 survey items. Participants aged under 18 years were also required to provide a signed digital consent form from a parent or guardian; if a parent or guardian was unable to sign the form, verbal consent was obtained via phone call.

Participants attended a single workshop lasting approximately 6 h, with 2–4 facilitators comprising suicide prevention researchers (some additionally with a clinical background) and a communications professional. Workshops held in partnership with a youth organisation sometimes also included a staff member from that organisation. After introductory activities, the workshop comprised three components, informed by the Double Diamond model [35], which involves exploring an issue broadly (divergent thinking) then taking action in a focussed manner (convergent thinking):

1. **Learn and define:** This didactic session focused on understanding, connecting, and learning. A facilitator overviewed benefits and challenges of social media in relation to self‐harm and suicide, and #chatsafe guidelines, highlighting safe communication practices illustrated with examples of safe language and social media content.
2. **Design:** In this session, young people conceptualised, collaborated, and created ideas for new #chatsafe social media content. This involved brainstorming activities and time to create and refine their prototyped ideas, generally in small groups.
3. **Reflect:** During this session, young people shared their designs and creations with the wider group. Young people and facilitators also engaged in a reflective discussion regarding the experiences of the design process, and the topics discussed during the day. Workshops ended with a ‘cool down’ activity.

Participants were paid an honorarium calculated at AUD $30/hour via bank transfer. They were sent a link to a quantitative T2 (post‐workshop) survey 1–2 days after completing the workshop and were sent one reminder a week later if they had not yet completed the survey. Facilitators attended debriefing meetings to reflect on workshop progress, content created, and any changes to be made to future workshops. The team then discussed how ideas generated from each workshop could be translated into content for the upcoming campaign.

### 2.6 Translation of Ideas

Campaign content was finalised by #chatsafe's Communications Advisor with input from other facilitators. Content was integrated into a campaign directly if it was finalised within the workshop, aligned closely with evidence‐based #chatsafe messaging, contained accurate and up to date information, and the #chatsafe team agreed it was appropriate during debriefing processes. Some content was adapted by the #chatsafe Communications Advisor, such as refining text and adding #chatsafe branding. Other ideas were refined by young people directly, as some participants were invited to become volunteer content creators or to attend a subsequent filming day (a brief content creation workshop). Content was subject to a review process by a #chatsafe subject matter expert with qualification as a registered or provisional psychologist.

### 2.7 Data Analysis

Evaluation survey data were downloaded from REDCap and analysed using IBM SPSS Statistics version 27. Descriptive statistics were reported for feasibility and acceptability questions. Confidence items were totalled. Due to scores violating the assumption of normality, a paired samples, two‐tailed Wilcoxon signed‐rank test was run to compare confidence pre‐ and post‐workshop for anyone who attended a workshop. As the open text T2 responses were optional, brief, and designed to contextualise participants' closed‐ended survey ratings rather than provide in‐depth data, these data were summarised by one coder (EC). The coder read all comments, and using a predominantly deductive approach, organised data around the predefined evaluation domains of feasibility, acceptability, safety, and self‐efficacy. The coder noted recurring or emotionally resonant themes, and selected illustrative quotations. The coder also reviewed field notes, and transcripts from audio recordings, in a similar fashion to summarise participants' ideas for campaign content. Campaign reach and engagement data were sourced from Meta Platforms Inc (Instagram).

## 3 Results

### 3.1 Overview

Nine workshops were held between November 2023 and January 2025. Seven were held in Victoria, and two interstate (New South Wales and South Australia). Four workshops were held in collaboration with local youth organisations. One workshop was scheduled in collaboration with a youth organisation based in Queensland, but was cancelled due to recruitment difficulties. One brief follow‐up workshop (a filming day) was held with four workshop participants who were invited back for a content creation day to refine two of their ideas. Additionally, six youth content creators were recruited from the workshops to create and refine ideas independently, with support from the #chatsafe team.

There were 137 T1 pre‐workshop survey responses (i.e., workshop registrations), of whom 107 participants (78.1%) attended a workshop. Individual workshop size ranged from 9 to 15 participants. Of the six workshops aimed at priority populations, sample size for individual workshops was as follows: culturally and linguistically diverse young people (*n* = 9), young people with lived experience (*n* = 12), LGBTQ+ young people (*n* = 15), and school‐aged adolescents (*n* = 9, 12, 13 across three workshops respectively).

Demographic data are presented in Table 1. The mean age of participants at T1 was 19.7 years (SD = 2.7 years, range = 15–25 years). Priority populations were represented across the workshop series. One in six identified as trans and/or gender diverse, and 55.1% reported LGBQ+ sexual orientation; 29.9% spoke a language other than English at home and 54.8% reported being from a cultural or ethnic minority; 51.0% were school‐aged (15–18 years).

**Table 1:** Workshop participant demographic data from Time 1 (*n* = 107) and Time 2 (*n* = 87).

		T1		T2	
**Demographic**	**Category**	***n***	**%**	***n***	**%**
Gender	Male	25	23.4	18	20.7
	Female	64	59.8	56	64.4
	Non‐binary, genderqueer, or gender‐fluid	17	15.9	12	13.8
	Prefer not to say	1	0.9	1	1.1
Identifies as transgender or gender diverse	Yes	21	19.6	15	17.2
	No	74	69.2	62	71.3
	Questioning/unsure	5	4.7	4	4.6
	Prefer not to say	2	1.9	2	2.3
Sexuality	Heterosexual/straight	48	44.9	37	42.5
	Lesbian	10	9.3	9	10.3
	Gay	3	2.8	2	2.3
	Bisexual	10	9.3	9	10.3
	Pansexual	9	8.4	6	6.9
	Queer	20	18.7	16	18.4
	Questioning/not sure	7	6.5	7	8
	Not listed	5	4.7	4	4.6
	Prefer not to say	3	2.8	3	3.4
Main language spoken at home	English	74	69.2	60	69
	Other language	32	29.9	27	31
Cultural or ethnic group	African	8	7.3	4	4.5
	Asian	28	25.7	26	29.4
	Australian, European, NZ or North American	25	23	24	27.1
	Jewish	1	0.9	1	1.0
	Maori	1	0.9	0	0
	Middle Eastern	2	1.8	2	2.2
	Mixed heritage	11	11.8	9	10
	South American	7	6.4	1	1.1
	Not specified	24	22.3	21	24.1
Aboriginal or Torres Strait Islander identity	No	106	99.1	87	100
	Prefer not to disclose	1	0.9	0	0
Highest level of education	Year 7‐8	1	0.9	1	1.1
	Year 9‐10	5	4.7	4	4.6
	Year 11‐12	43	40.2	34	39.1
	Some university	31	29	24	27.6
	Some TAFE	7	6.5	5	5.7
	Completed bachelors degree	13	12.1	13	14.9
	Completed TAFE course/certificate	6	5.6	6	6.9
Employment status	Casual	40	37.4	37	42.5
	Part time	12	11.2	8	9.2
	Full time	1	0.9	0	0
	Studying and working	18	16.8	15	17.2
	Full time student	36	33.6	31	35.6
	Looking for work	33	30.8	24	27.6
	Unable to work	2	1.9	1	1.1
	Other	6	5.6	5	5.7
	Prefer not to disclose	1	0.9	1	1.1

*Note:* Columns may not add up to 100% due to missing data or where participants could select multiple items.

Additionally, a high proportion of attendees reported lived experience of suicide or self‐harm: 64.5% reported lifetime self‐harm thoughts, 43.9% lifetime self‐harm behaviour, 67.3% lifetime suicidal thoughts, and 26.2% lifetime suicide attempt. Just over one quarter (26.2%) reported ever experiencing the suicide death of someone they know, such as a friend or extended family member, with most bereaved participants (60.7%) reporting this death was within the last 3 years.

### 3.2 1 A. Feasibility, Acceptability and Safety of the #Chatsafe Workshop

#### 3.2.1 Feasibility

Eighty‐seven participants (81.3% of workshop attendees) completed the post‐workshop (T2) survey. As seen in Table 1, demographics were similar across both time points, though a slightly higher proportion of female participants and those employed on a casual basis completed T2. Responses to feasibility questions (perceived support, accessibility, and convenience) are presented in Figure 1 and Table S1. The mean rating across all items was 4.5/5 (SD = 0.1). Participants responded positively to feasibility items; for example, all participants (100%) agreed that they felt supported to participate in the workshop, and most agreed that their needs were met (94.3%), that it was convenient to participate (85.1%), and that they were remunerated fairly (93.1%). In relation to feasibility, open‐text feedback from T2 was positive, with participants endorsing workshop facilitation, locations, and the payment associated with attendance (described as ‘very fair’‐ Woman/female, age 22).

**Figure 1:**
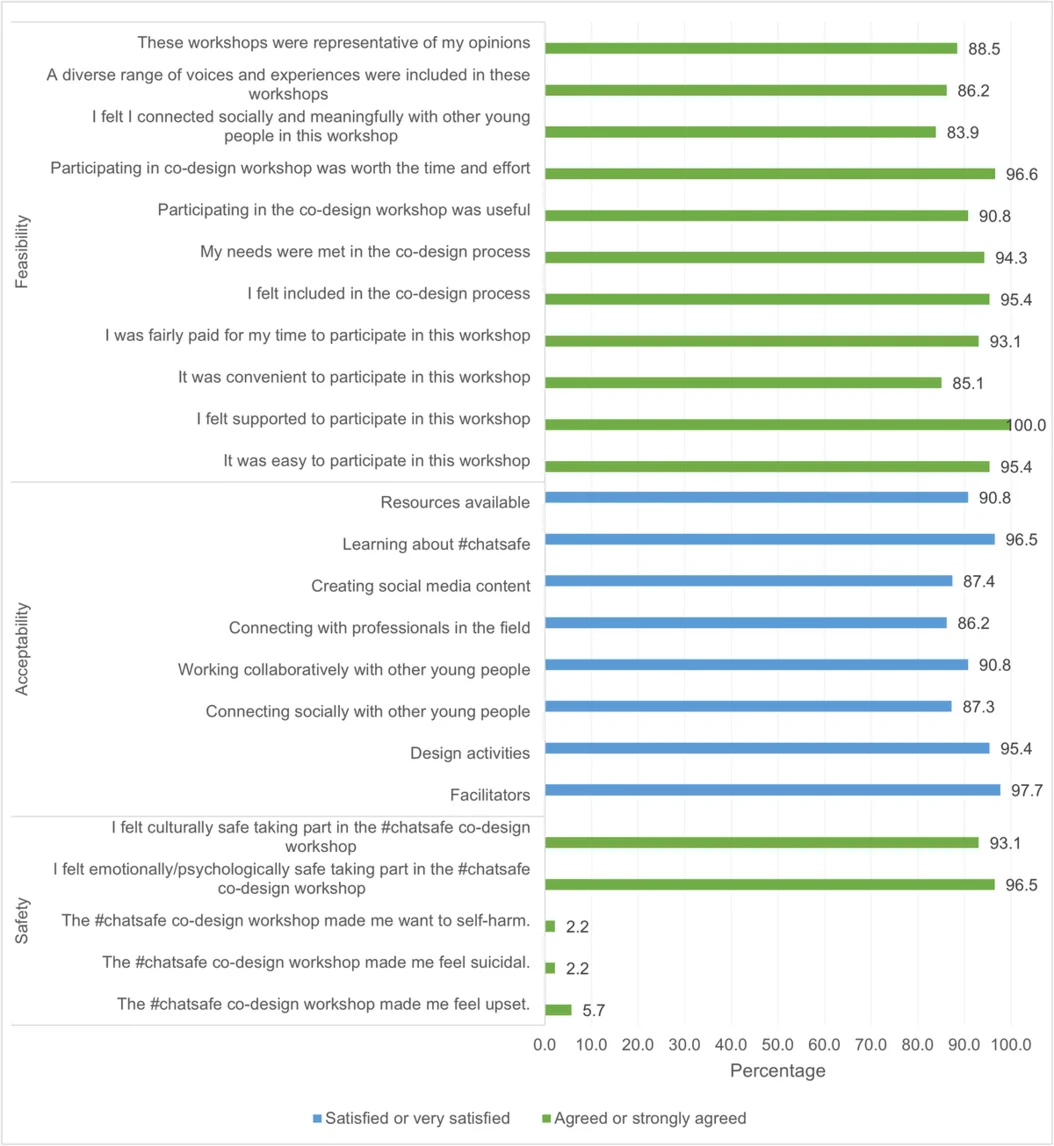
Time 2 workshop feedback questions (*n* = 87).

Minor criticisms related to certain practical aspects of the workshops, such as technical difficulties (e.g. uploading prototypes from their phones to a shared drive for viewing), the location being a long distance to travel, or not catering sufficiently for those with certain dietary requirements. Others made suggestions to improve the structure of the day, such as shortening the educational component (Learn and Define) and incorporating more interactive activities as ‘sitting through long explanations was tiring’ (Woman/female, age 22).

#### 3.2.2 Acceptability

Overall, 94.3% of participants reported being satisfied or very satisfied with the workshop, and 96.5% reported mostly enjoying the workshop, or enjoying it all. Responses to additional acceptability items are presented in Figure 1 and Table S2.

Open‐text feedback from T2 was generally favourable and consistent with the quantitative findings. For example, several commented that other young people in attendance – most of whom were strangers prior to the workshop – were friendly and made them feel welcome, ‘form[ing] meaningful connections’ (Woman/female, age 18) whilst being ‘exposed to different perspectives’ (Man/male, age 16). Views on workshop demographics were mixed; some appreciated diverse perspectives, whereas others (particularly in a workshop with many psychology undergraduates) wanted greater diversity and suggested stratifying workshops by age:

Majority of the people in the group were younger than myself and had different opinions and or issues in their life that wouldn't be super relevant to someone in their 20's.

– Woman/female, age 22

Participants typically praised the creation activities that occurred during the ‘Design’ component of the day, with several commenting that this component was fun. Some described enjoying the opportunity to ‘tap into [their] creative side’ and ‘enjoyed collaborating with like‐minded peers’ (Woman/female, age 15). Some appreciated the less structured nature of this section, as the facilitators ‘offered guidance on the process but didn't dictate what we could and couldn't do…. I appreciated how much they approached with curiosity over judgement and sought to understand our perspectives’ (Woman/female, age 21). However, others commented that this section could have been improved by provision of more ‘tips on creating content (such as what software to use)’ (Man/male, age 18), seeing examples of content created by previous workshop participants, and more time to refine their ideas as some prototypes ‘felt a bit rushed and unfinished’ (Woman/female, age 22). Some expressed preference to ‘focus on quality of content over quantity’ (Man/male, age 17). A minority commented on their desire to iterate their ideas further, to see the final results of the campaign, and to contribute in a more meaningful manner beyond a single workshop:

Good co‐design to me means being aware of and celebrating the final product, rather than doing one workshop and never hearing about the project again.

(Non‐binary participant, age 23)

#### 3.2.3 Safety

As reported in Figure 1 and Table S3, most participants reported feeling emotionally and psychologically safe (96.5% agreement) and culturally safe (93.1% agreement) during the workshop. A minority reported that the workshop made them feel upset (5.7% agreement) and two participants (2.2% agreement) reported that the workshop caused them to feel suicidal or like they wanted to self‐harm. Inspection of individual response patterns indicated that, despite these responses, both participants reported positive feedback on the workshop, including agreeing that the workshop was enjoyable and that they felt supported. These two participants were displayed a question asking if they wished to stop the survey and withdraw; neither endorsed this option. No adverse events were identified during workshops and the on‐call psychologist was not needed at any point. No safety concerns were raised in open‐text comments. Many participants praised the workshops for having a ‘really welcoming environment’ (Woman/female, age 18) that they perceived as safe, inclusive and ‘stigma free’ (Woman/female, age 20). Furthermore, several participants praised the provision of sensory toys and a quiet space, with some participants being ‘thankful that they were provided’ (Non‐binary participant, age 19) even if a participant chose not to use them.

I felt safe and welcome within the environment. I felt I could talk about my experiences without fear of judgement and I got the opportunity to listen to others experiences and expand my knowledge on how to help people.

(Non‐binary participant, age 19)

#### 3.2.4 1B: Self‐Efficacy

Table 2 presents items pertaining to confidence in communicating safely online about self‐harm and suicide. There was an increase in confidence across all items between T1 and T2. A Wilcoxon Signed Rank Test indicated a statistically significant increase in confidence following participation in a #chatsafe workshop, *z* = −5.99, *p* < 0.001, with a medium effect size (*r* = 0.43) [41]. The mean score on the confidence items increased from 22.3 (SD = 4.5) to 24.9 (SD = 3.7) post‐workshop. Furthermore, 78.2% agreed that the workshop led to improved knowledge about safe online communication about self‐harm and suicide, and 81.6% indicated that the workshop improved or strongly improved their ability to communicate safely online about self‐harm and suicide.

**Table 2:** Confidence in communicating online about suicide at Time 1 and Time 2.

	Time 1 (*n* = 105)		Time 2 (*n* = 86)	
Confidence Item	*M*	*SD*	*M*	*SD*
Having a conversation about self‐harm or suicide	3.98	0.94	4.13	0.68
Starting a conversation about self‐harm or suicide	3.38	1.18	3.78	0.87
Using safe language when talking about self‐harm or suicide	3.71	0.93	4.33	0.73
Recognising harmful content about self‐harm and suicide	4.14	0.80	4.40	0.76
Responding to self‐harm and suicide content online	3.31	1.10	4.05	0.88
Reporting self‐harm and suicide content online	3.73	1.13	4.27	0.90

In open‐text items, participants highlighted educational messages that resonated with them, including safe language, recognising and reporting unsafe content, and the importance of talking about self‐harm and suicide in a responsible manner. A minority commented that they would have liked more information about direct conversations about suicide with other people. Regardless, most participants felt they learned something new:

I feel that my understanding of the need for safe online communication was especially improved… I learned some very helpful places to go for extra support.

(Woman/female/non‐binary participant, age 22)

### 3.3 2 A. Insights From Participatory Activities and Content Outputs

Participants created prototypal content both in groups and individually, using various formats, including drawing storyboards, photographs, stop motion animation, audio recordings, digital graphic design, and filming short‐form videos. They shared suggestions around various formats for sharing #chatsafe's educational messages, including carousel‐style social media posts with static text, narrative‐driven videos and animations, and humorous or relatable memes. Examples of content created by young people during the workshops are presented in Figure 2.

**Figure 2:**
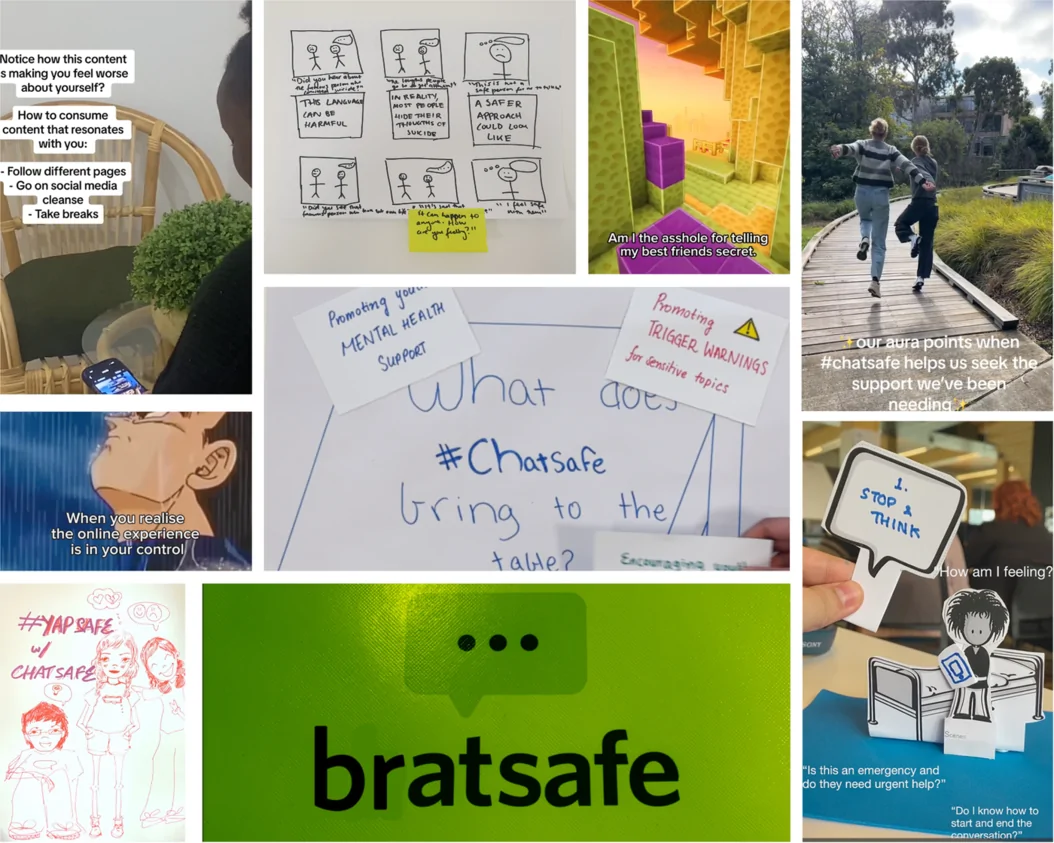
Example content created by young people during workshops, including prototype storyboards, memes, and stills from short form videos.

Young people emphasised that content needed to be relatable, relevant and engaging. They highlighted the oversaturation of certain types of social media content they are routinely exposed to, including mental health‐related messaging, and that #chatsafe content needed to be perceived as youth‐friendly, authentic and important. They noted that they would be more inclined to take advice from health promotion accounts, or health professionals, if advice was perceived to be relevant, qualified and evidence‐informed. They emphasised keeping any text‐based messaging simple and readable.

They also recommended the occasional use of humour, social media trends (particularly emphasised in workshops with school‐aged young people), and moments of lightness when discussing suicide prevention. Despite the sensitive nature of the topic, they felt that these elements helped make the content more attention‐grabbing and engaging, provided this was done with care. For example, some participants created video content using trending sounds and music, dance moves, and memes. Participants also identified that more traditionally educational content is still important, though it may not have the mass appeal of lighter content. They advised that videos featuring real people – ideally young people – is particularly engaging and perceived to be authentic.

### 3.4 2B. Reach and Engagement of #chatsafe Edition 2 Social Media Campaigns

Content directly informed or created through the #chatsafe workshops was first incorporated into a targeted, paid Instagram campaign, including a range of short‐form videos featuring young people and #chatsafe team members, static carousel posts, and memes (see toolkit – Appendix 3 – for examples). The campaign targeted location (Australia) and age (13–25). Across 12‐weeks in 2024, the campaign reached 950,000 young people, approximately 29% of the Australian population in this age group. The campaign successfully reached and engaged young people across all age groups, genders and regions. For every account of a young person reached, content was seen on average nine times. The campaign also had over 300 shares, more than six times the number of shares #chatsafe typically achieves on average per month. Content directly informed by insights from the #chatsafe workshops performed particularly well; for example, pop culture memes tied with relevant suicide prevention captions had the highest number of reactions. Other high performing content included an educational carousel explaining why the term ‘kms’ (kill myself) can be problematic, a video featuring drawings and narration from a youth content creator about the nature of safe social media content, and short videos featuring members of the #chatsafe research team verbally explaining aspects of the #chatsafe guidelines.

Insights from the workshops also informed a second paid #chatsafe Instagram campaign focused on school‐aged young people, aged 13–19 years, which ran over 6 weeks in early 2025. For this campaign, content was intentionally ‘lighter’ in tone while remaining aligned with #chatsafe principles, and incorporated a greater proportion of content generated by young people during school‐aged workshops (including several short‐form videos featuring workshop attendees using social media trends). This youth‐generated content was interspersed with content developed by the #chatsafe team. Campaign content focused on themes that resonated most with teenage participants, including understanding suicide stigma, guidance on safe language, supporting friends, and ways to get support. The campaign reached 818,000 young people aged 13–19, approximately 54% of the adolescent population in Australia, successfully reaching all genders and regions, and over 9100 reactions. High performing content included informative carousels created by the #chatsafe team, as well as meme and trend‐based content that was youth‐made.

## 4 Discussion

Overall, the evaluation identified high levels of workshop feasibility, acceptability, and safety of the #chatsafe Model across nine workshops. Insights informed two wide‐reaching #chatsafe social media campaigns, which in combination reached nearly two million young people across Australia. Overall, the findings indicate that the #chatsafe Model is a valuable and safe approach for meaningfully involving young people in suicide prevention social media campaigns, building on early #chatsafe participatory research [15] and extending the evidence base for youth‐informed interventions in suicide prevention. These results also support the implementation of the #chatsafe Model, and the safety of youth participation in suicide prevention research more broadly [18, 21].

Many participants were from groups historically under‑represented in suicide prevention research [4], including over 60% with lived experience of self‑harm, suicidal thoughts and behaviours or bereavement by suicide, more than half identifying as LGBTQ + , and substantial numbers from culturally and linguistically diverse backgrounds and aged under 18 years. Some of this success may be attributed towards partnering with other youth organisations for recruitment, including organisations that work with these specific communities (e.g. multicultural organisations), facilitating trust and engagement, and reducing anxiety or uncertainty about participation [24]. We trialled a mix of targeted workshops, and workshops open to a wider audience. Targeted workshops may be necessary for creating tailored content, but may also allow participants to feel more comfortable by allowing them to work with people of a similar demographic, maximise cooperation, and reduce the risk of unequal power dynamics [24, 42]. Regardless, the data suggest that the model is both acceptable and effective in reaching several priority audiences.

Feedback data indicated that the workshops were safe, supporting prior research into the overarching safety of suicide prevention research [4, 16, 18, 19]. Even though no safety issues were identified during the workshops, two participants reported the workshop made them feel suicidal; their other feedback was positive, suggesting a straight‐lining response style and possible error. Regardless, as six per cent reported feeling distressed after the workshop, this highlights the importance of robust safety procedures, including preventative as well as responsive strategies built into the #chatsafe Model.

Although participants' data was positive regarding feasibility, it is important to acknowledge some of the process‐related challenges identified by workshop facilitators. The workshops were resource intensive, requiring substantial staff time, planning, financial investment, and relationship‐building with community partners. This aligns with previous research that has described the significant planning and costs associated with meaningful participatory research [24, 25, 43]. During debriefing sessions, facilitators – despite the majority being experienced suicide prevention researchers, and some having clinical training – also reported that sessions could sometimes be emotionally demanding, particularly when working with populations significantly affected by suicide and when workshop discussions had a greater focus on their lived and living experiences. This highlights the need for robust facilitator training and support, careful preparation, debriefing, and ongoing supervision. Furthermore, despite initial intentions to run workshops across a broader range of locations across Australia, logistical and resource constraints meant that workshops were primarily conducted onsite.

Workshop procedures were also refined by staff over time, guided by ongoing review of open‐text evaluation data and facilitator meetings. Refinements included streamlining registration processes, revising plain language statements and consent forms to improve clarity, and making greater use of text message reminders. We also modified the didactic ‘Learn and Design’ component of the workshop to reduce its length, incorporate more interactive elements (such as small group discussions), and further tailor content to different participant groups (such as adjusting messaging to be more age‐appropriate for school‐aged workshops). For some workshops, greater emphasis was placed on trialling a range of content ideas rather than striving for a single ‘perfect’ prototype, which appeared to reduce performance pressure and ultimately produced more usable content. Providing participants with more time and autonomy to develop and refine their ideas, with less facilitator intervention, further enhanced creativity and ownership. However, sometimes this resulted in participants prototyping ideas that were not closely aligned with #chatsafe messaging; facilitators thereby sought to strike a balance between providing guidance and allowing participants to work autonomously. Finally, considering participant feedback about the challenges refining their ideas within a single workshop, we piloted a shorter, follow‐up content creation workshop where interested young people attended a subsequent filming day (a tenth workshop, that was not formally evaluated) to refine their original concepts. Collectively, these adaptations demonstrate the value of treating youth participation as a dynamic and flexible process [44] and suggest that researchers remain responsive to feedback.

The workshops generated nuanced insights into young people's preferences for suicide prevention messages on social media. Participants reinforced and extended findings from previous co‐design work [15], including the importance of authenticity and relatability, and provided detailed guidance on tone, format, and narrative style. For example, they highlighted the role of humour and ‘lightness’ in helping some messages cut through the overwhelming volume of content in young people's social media feeds, provided that these elements are used sensitively and without trivialising distress or suicide. Although using humour as part of suicide prevention messaging is generally not recommended [29], and there is limited research into safe use of humour in this context, this highlights that humour can resonate with some young people even in this context [45]. Participants also emphasised the importance of seeing relatable content, featuring and made by young people, that can bridge the gap between formal health messaging and young people's everyday online experiences, potentially enhancing credibility and impact.

The campaigns developed through workshop outputs performed strongly. A reach‐to‐impression ratio of 9:1 indicates that young people were exposed to #chatsafe messages on average nine times, which is promising in terms of reinforcement, recall, and potential behaviour change [46]. Moreover, content derived from the workshops was shared more frequently than #chatsafe's usual ‘always‐on’ social media content, and overall sharing rates were higher than for previous campaigns. In the context of a crowded and often confusing online environment, this willingness to share implies that young people found the content trustworthy, meaningful, and worth recommending to their peers. However, the impact of viewing these youth‐informed campaign materials (including the use of humour) is currently unknown.

We identified significant improvements in participants' confidence regarding safe online communication post‐workshop. Most participants also reported learning something new, suggesting that the #chatsafe workshops can deliver dual benefits: generating high‐quality, youth‐informed content while also building participants' skills and confidence. These findings are notable, given that the primary mechanism for educating young people within the #chatsafe programme to date has been exposure to educational social media content [30]. The active, dynamic participatory workshop process may produce stronger improvements in safe communication compared to passive exposure to campaign content alone through higher elaboration and critical thinking [47]. Future research should examine the relative impact of ‘passive’ exposure to social media campaign content with in‐person educational activities, particularly for topics as complex and sensitive as suicide prevention.

### 4.1 The #chatsafe Youth Participation Toolkit

To support other researchers, practitioners, and communications professionals, a #chatsafe Model toolkit has been made available for free download as an appendix (Appendix 3). The toolkit documents the workshop structure and key processes in a replicable way. It also incorporates learnings from this evaluation. For example, it includes enhanced guidance on when and how to ‘step up’ the participatory ladder [48, 49], creating opportunities for young people to be more involved beyond initial brainstorming and prototyping. Although developed in the context of suicide prevention, the toolkit is designed to be relevant for broader range of mental health and wellbeing initiatives, where youth perspectives are important to the success of a project.

### 4.2 Limitations

Limitations of this study have been identified. First, the evaluation relied on self‐reported data, including brief open‐text responses. Second, at the time of data collection there was no validated measure of confidence for communicating safely about suicide online; consequently, bespoke but non‐validated items were used. A validated tool is now available [50] for future evaluations in this context. Third, the study did not include long‐term follow‐up, so we cannot determine whether confidence was maintained over time or whether participants subsequently applied the skills learnt. Fourth, despite efforts to recruit broadly, most workshops were held locally; this may limit generalisability to other regions. Fifth, this study was only able to assess the reach and engagement associated with campaign materials created throughout the study, not the impact of viewing the campaign on the target audience. Research into social media‐based suicide prevention interventions is limited [30]. A manuscript is being prepared for publication which will present an impact evaluation of #chatsafe content conducted via a randomised controlled trial. Finally, although we successfully engaged several priority groups, some populations, such as Aboriginal and Torres Strait Islander young people, were not adequately represented in this phase of the work [34]. Given the distinct cultural, historical, and social determinants of suicide in these communities, dedicated, culturally informed co‐design processes are required to apply the #chatsafe model to these communities, and will likely require further adaptation and involvement beyond a single workshop.

## 5 Conclusions

The results of this evaluation indicate the #chatsafe model is highly feasible, acceptable, and safe for a diverse range of young people, albeit requiring significant organisational resources and buy‐in. The model appears to offer mutual benefits: producing impactful, shareable social media campaigns while simultaneously enhancing participants' confidence in communicating safely about suicide online. The results support the integration of structured youth participation processes into the development of digital suicide prevention interventions. Future research may explore whether participation in #chatsafe workshops leads to broader benefits, such as improvements in suicide literacy, reduced suicide‐related stigma, and enhanced help‐seeking intentions and behaviours. Researchers may also explore application of the #chatsafe Model to other youth participation activities, both within and beyond suicide prevention, and adapt the model to other target populations.

## Author Contributions

**L. La Sala:** conceptualisation, funding acquisition, investigation, methodology, supervision, writing – original draft, writing – review and editing. **E. R. Carrotte:** data curation, formal analysis, investigation, project administration, writing – original draft, writing – review and editing. **C. Cooper:** conceptualisation, investigation, writing – original draft, writing – review and editing, methodology. **M. Lamblin:** conceptualisation, funding acquisition, investigation, methodology, project administration, supervision. **E. Brown:** methodology, writing – review and editing. **J. Robinson:** conceptualisation, funding acquisition, methodology, supervision, writing – review and editing.

## Ethics Statement

Study procedures were approved by The University of Melbourne Human Research and Ethics Committee (ID: 26610). All participants signed a digital consent form via REDCap, and a parent/guardian completed an additional consent form if a participant was aged younger than 18 years. All workshop participants signed an assignment of rights form. The content created during the #chatsafe workshop is thereby owned by Orygen, for its distribution and adaptation in print and other media.

## Conflicts of Interest

The authors declare no conflicts of interest.

## Supporting information

List of Appendices

**Supporting File 1**.

Supporting File 2

Supporting File 3

## Data Availability

The data that support the findings of this study are available from the corresponding author upon reasonable request.
