# Supplementary material for: The #chatsafe Youth Participation Model: Evaluating Participatory Workshops Involving Young People in the Design of Suicide Prevention Initiatives
Source: Health Expect. 2026 Oct 3;29(5):e70885. doi: 10.1111/hex.70885 (PMC13633697; doi:10.1111/hex.70885)
Supplement: Supplementary file 1 [file HEX-29-e70885-s003.pdf]

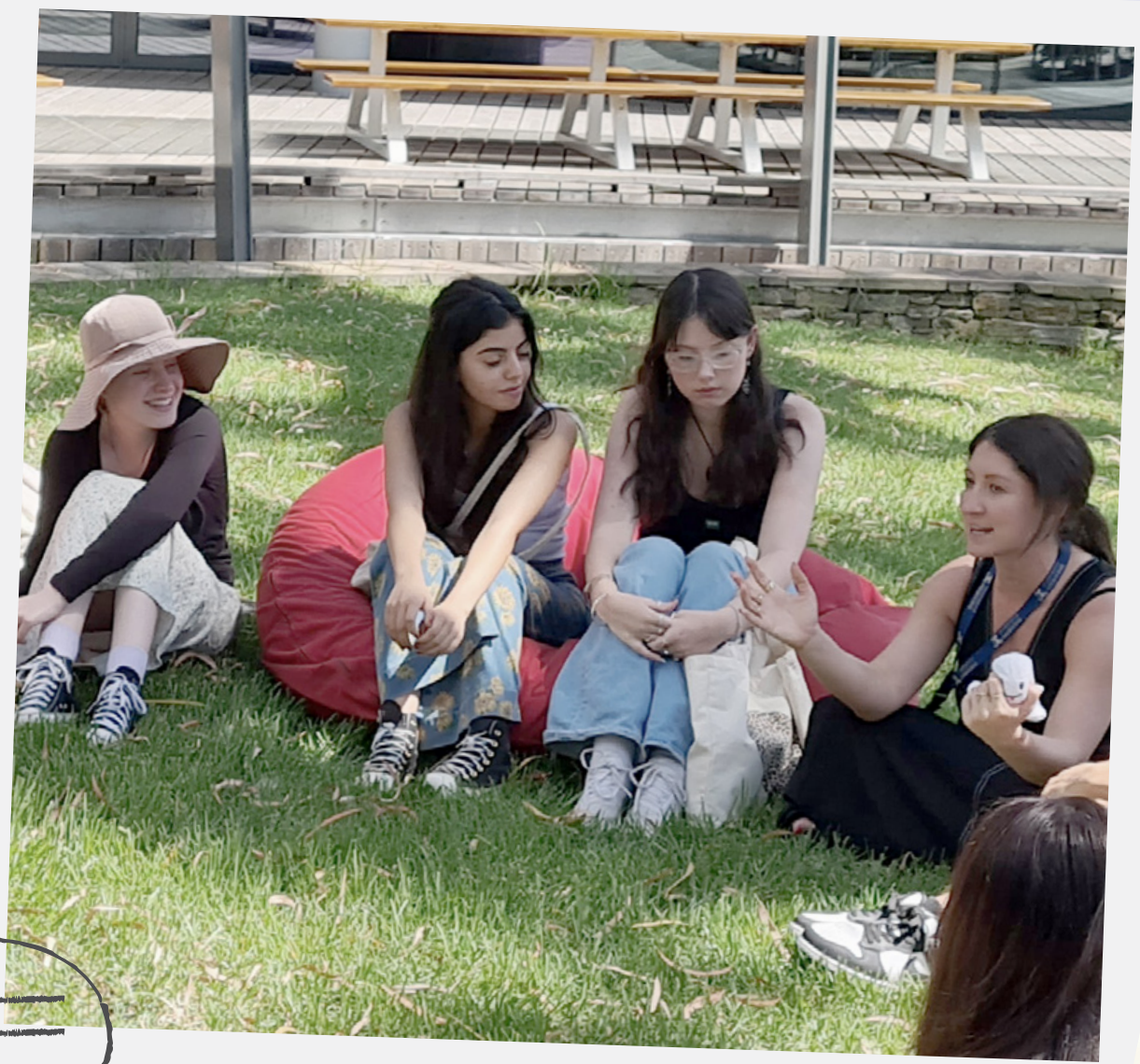

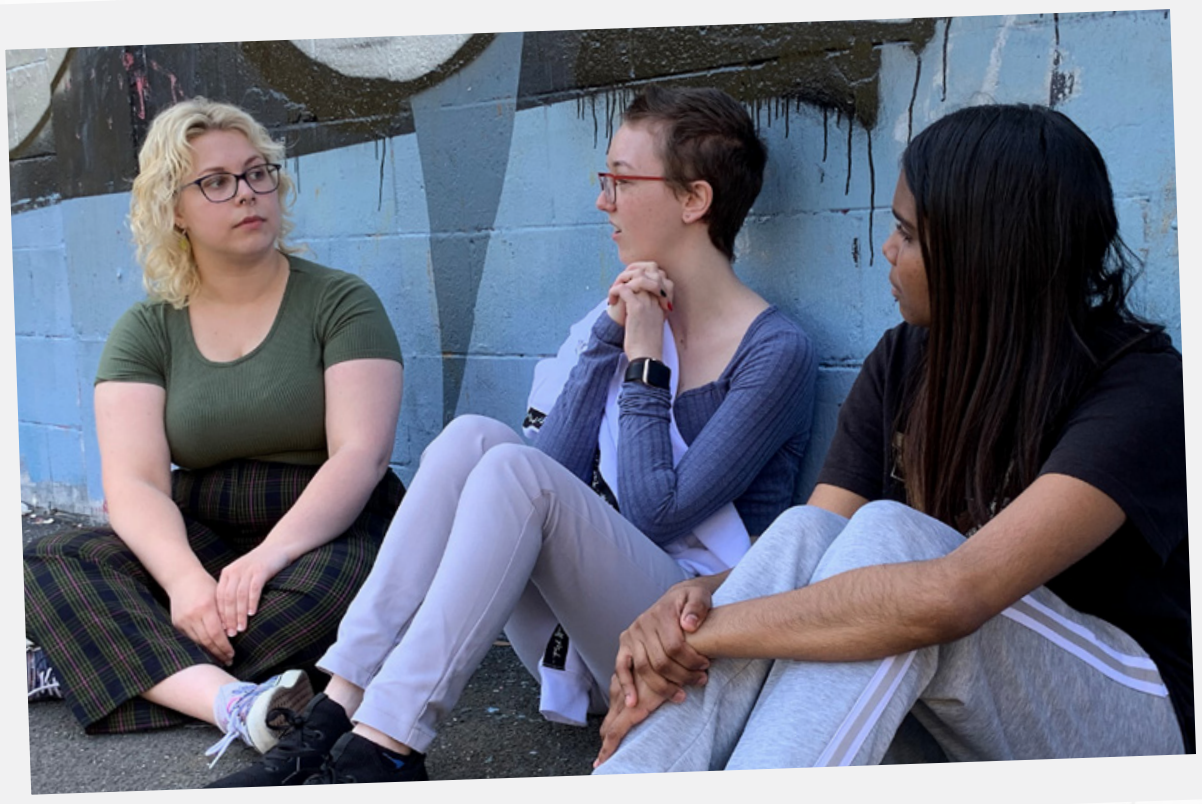

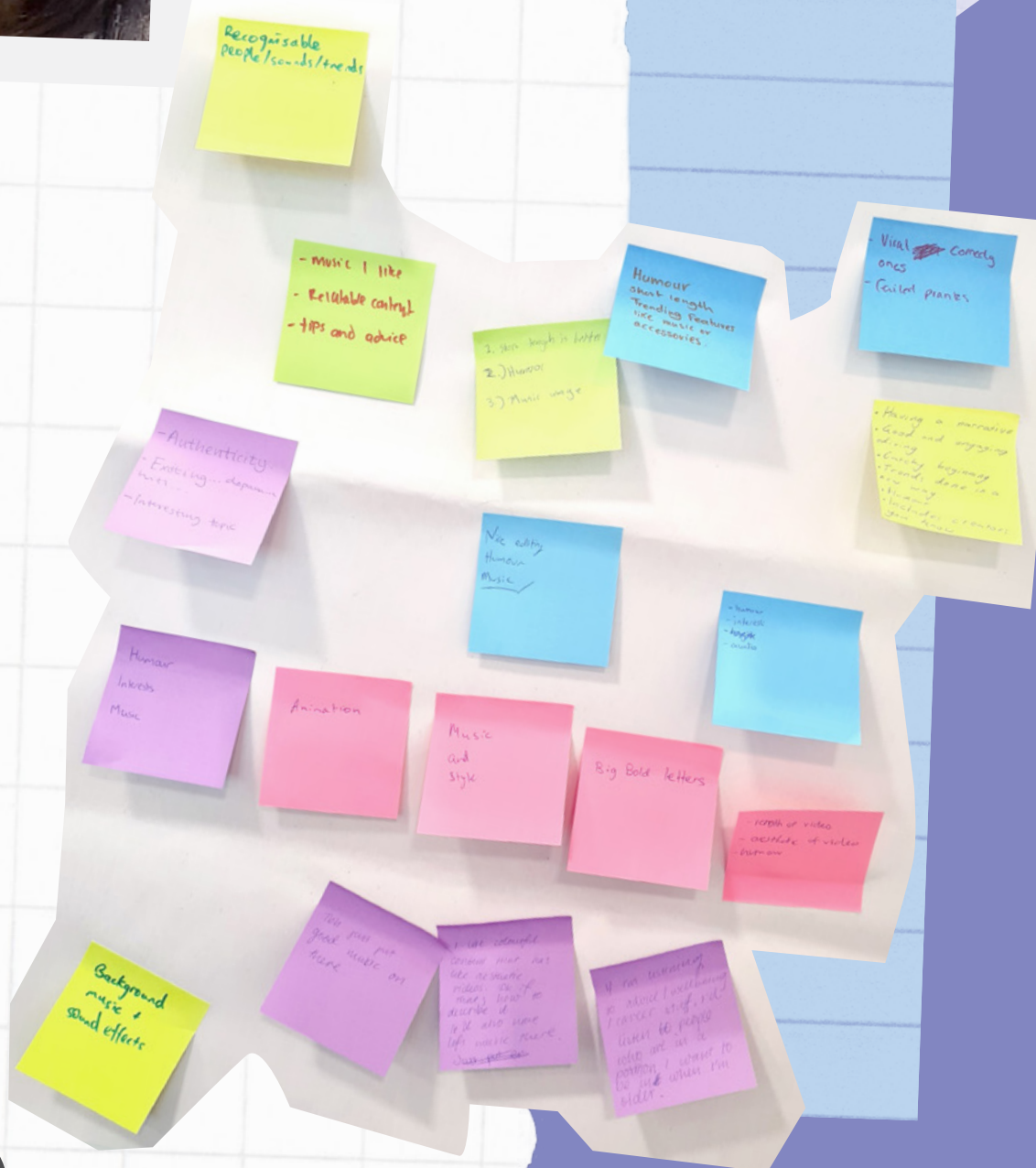

#chatsafe Youth Participation Model

A step-by-step toolkit for running effective and engaging creative design workshops with young people

#chatsafe®

orygen

Acknowledgements
This work was primarily conducted on the lands of the Wurundjeri people of the Kulin Nation. Orygen acknowledges the Traditional Owners of the lands we are on and pays respect to their Elders past and present. Orygen recognises and respects their cultural heritage, beliefs and relationships to Country, which continue to be important to the First Nations people living today.

The #chatsafe team extends its gratitude to all young people involved in #chatsafe participatory workshops, and to our community partners who made the workshops possible. We also thank all workshop participants who assisted us to develop this toolkit through providing their suggestions and recommendations.

Suggested citation
La Sala, L., Carrotte, E. R., Cooper, C., Lamblin, M., Brown, E., & Robinson, J. (2026). The #chatsafe Youth Participation Model: evaluating participatory workshops involving young people in the design of suicide prevention initiatives. Health Expectations, e70885. doi: 10.1111/hex.70885

Contents

3 Contents

5 Overview

7 Planning cycle

8 Step 1. Planning and resourcing

13 Step 2. Recruitment and consent

18 Step 3. Holding the workshop

25 Step 4. Reflection and feedback

26 Step 5. Translating workshop ideas into actions

28 Step 6. Closing the loop

29 Case study: #chatsafe social media campaign

34 References

34 Resources

35 Appendix: Wellness plan template

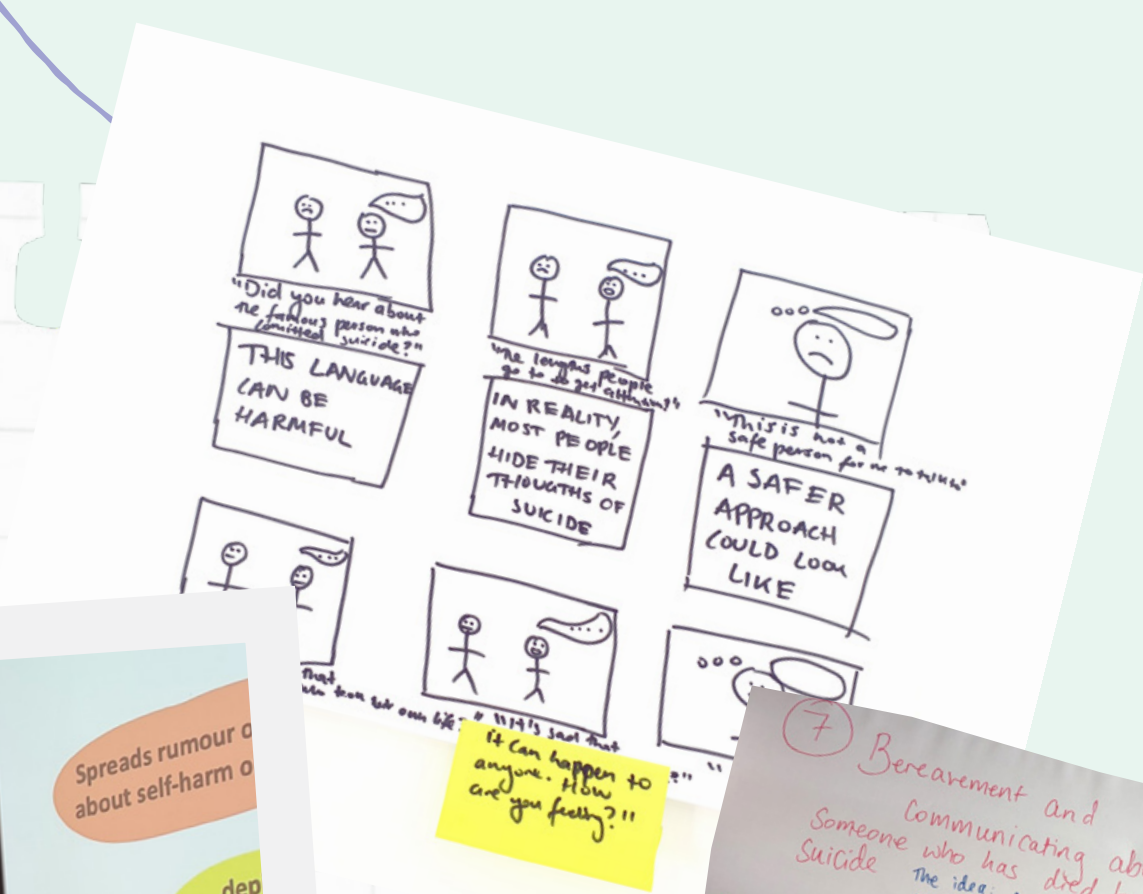
THIS LANGUAGE CAN BE HARMFUL
IN REALITY, MOST PEOPLE HIDE THEIR THOUGHTS OF SUICIDE
A SAFER APPROACH COULD LOOK LIKE

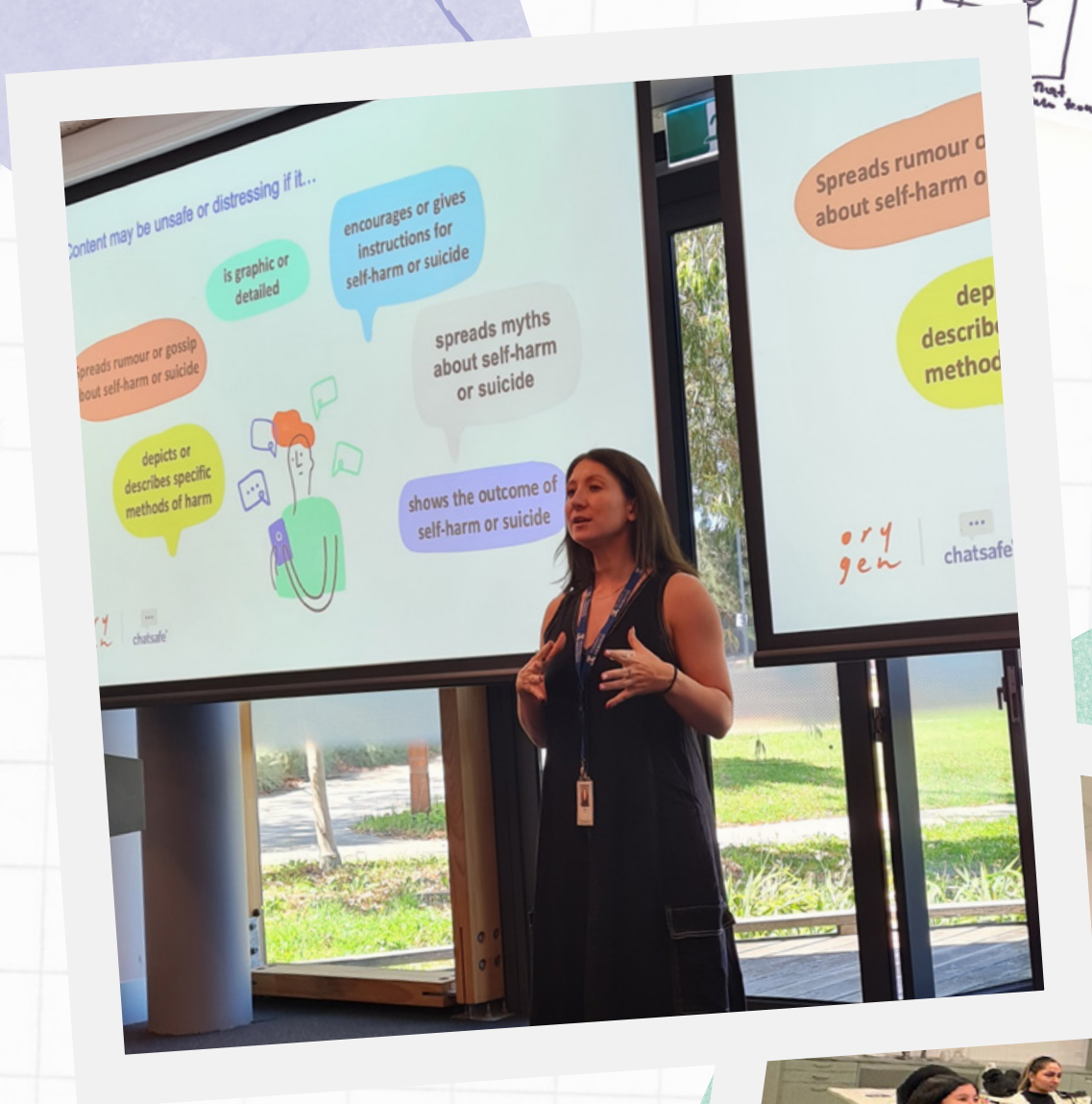
is graphic or detailed
encourages or gives instructions for self-harm or suicide
spreads myths about self-harm or suicide
depicts or describes specific methods of harm
shows the outcome of self-harm or suicide
chatsafe

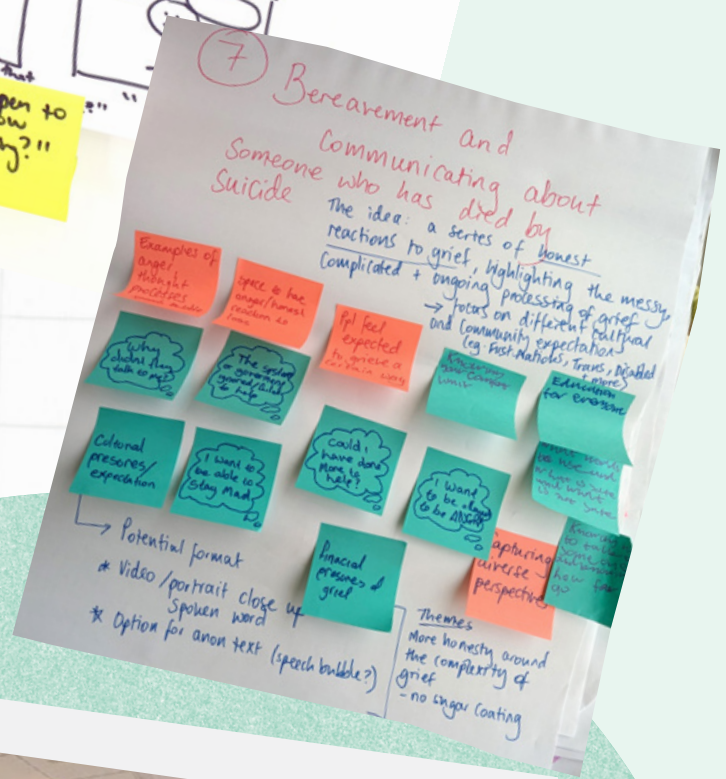
Bereavement and communicating about someone who has died by suicide

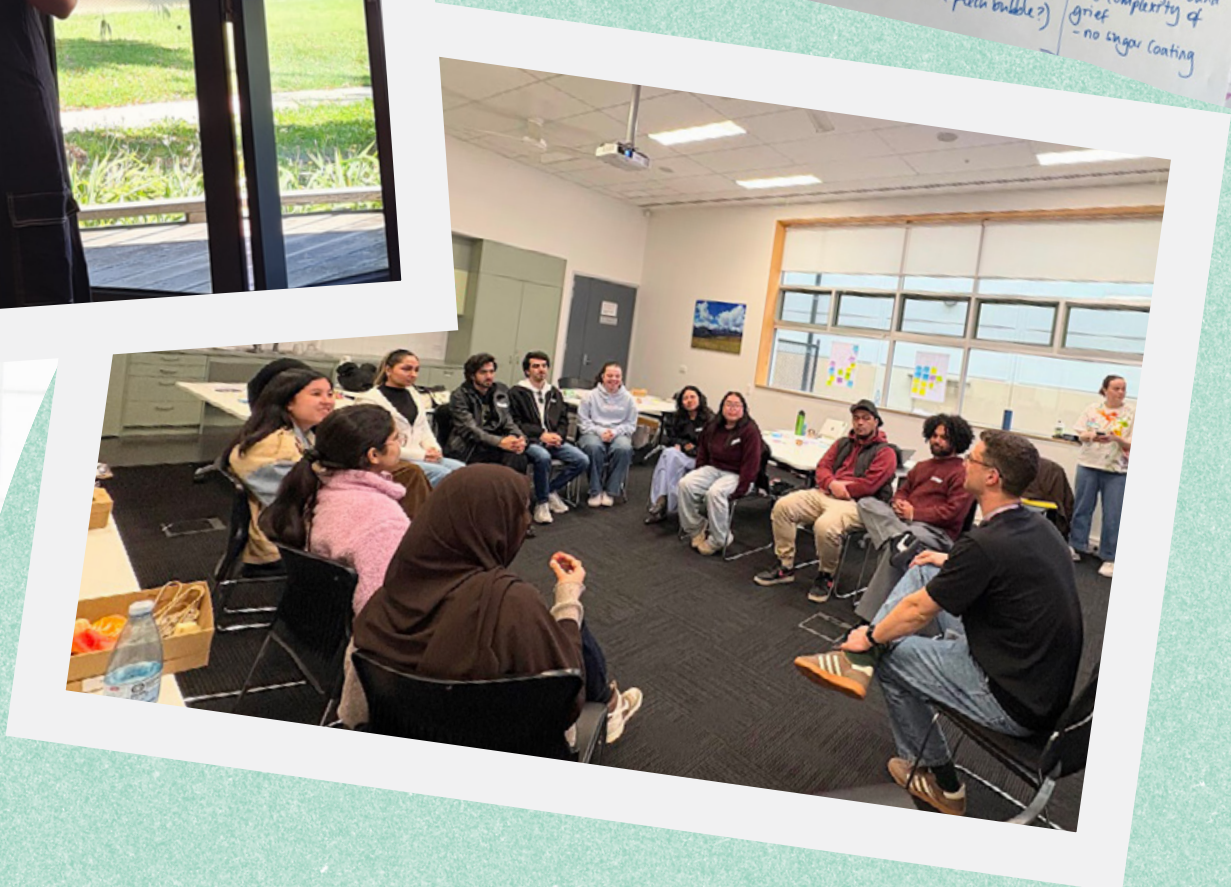

Overview

Introduction to the #chatsafe Youth Participation Model

Despite a large field of research outlining the importance of participatory research and its underlying values, some researchers have reported not knowing where to start, or which models and activities work well for involving young people.

It's becoming **more common to include young people** in the design and implementation of new content, products, interventions and services that are aimed at a youth audience. It is also becoming more common to involve young people in projects that relate to sensitive topics, including suicide prevention research activities (Webb et al., 2023), though this is less common than other fields.

The **#chatsafe Youth Participation Model** ('#chatsafe Model') is designed for projects involving young people aged 15-25 years in creative design activities.

While the model was designed for suicide prevention activities specifically, our goal was to create something that could be used across many settings, fields, and topics -- including other topics that may be sensitive or associated with psychological distress.

This document ('**the toolkit**') describes how researchers, designers, and others can apply the #chatsafe Model.

This toolkit will talk you through ways to:

1. effectively plan and prepare for participatory workshops with young people
2. educate young people on the relevant topic of your work
3. help young people brainstorm creative ideas
4. work with young people to bring their ideas to life.

It also includes a case study that illustrates the #chatsafe Model in action.

The #chatsafe Model was created and evaluated by the #chatsafe team at Orygen, the National Centre of Excellence in Youth Mental Health, and Centre for Youth Mental Health at the University of Melbourne.

Tip

You can also use the #chatsafe Model to:

- create content for a social media campaign
- inform a digital mental health resource or intervention
- design a physical space e.g. a waiting room in a youth health service
- design a youth-friendly app, video or podcast

and much more...

#chatsafe partners and collaborators around the world can use this toolkit to run #chatsafe workshops.

The participatory ladder

Many youth participation frameworks refer to a **participatory ladder**. In this context, a participatory ladder describes different levels of youth participation across the course of a project.

At the bottom of the ladder, there is no participation at all, or only tokenistic participation. At the top of the ladder, young people initiate and lead activities, or are heavily involved in other ways.

The #chatsafe Model falls in between these two extremes, and most existing participatory frameworks would describe our main workshop approach as either 'consultation' or 'engagement'. It is best suited to contexts where you are seeking **a lot of ideas from a large group of people** through workshops, which can later be iterated and refined (predominantly by a research or media team).

The level of engagement suitable for your project will depend on your resources and aims.

The #chatsafe Model is designed to be **flexible**. We encourage you to adapt this framework to step up the ladder, if this is possible and appropriate for your project.

Stepping up the ladder involves giving young people more power and ownership over the creative design and the project implementation. For example, this might look like:

- involving young people throughout the research and design process, such as setting priorities around the workshops themselves
- involving young people in decision-making, such as including polls or group consensus activities in workshops
- inviting young people back into a future workshop to present refined ideas and seek feedback
- invite interested young people to ongoing creative or advisory roles
- involving young people as facilitators of workshops

See the case study on page 29 for an example.

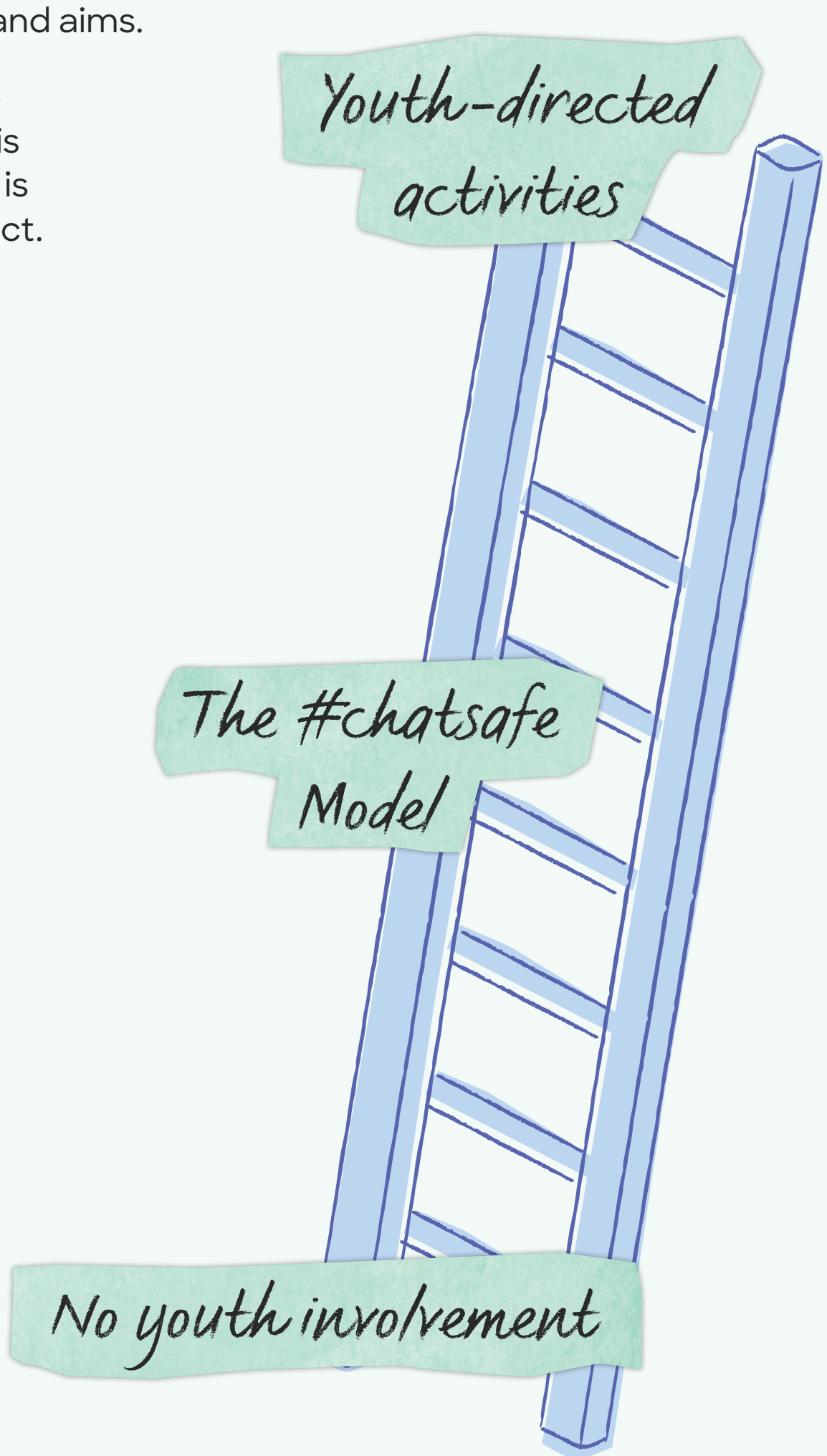

Planning cycle

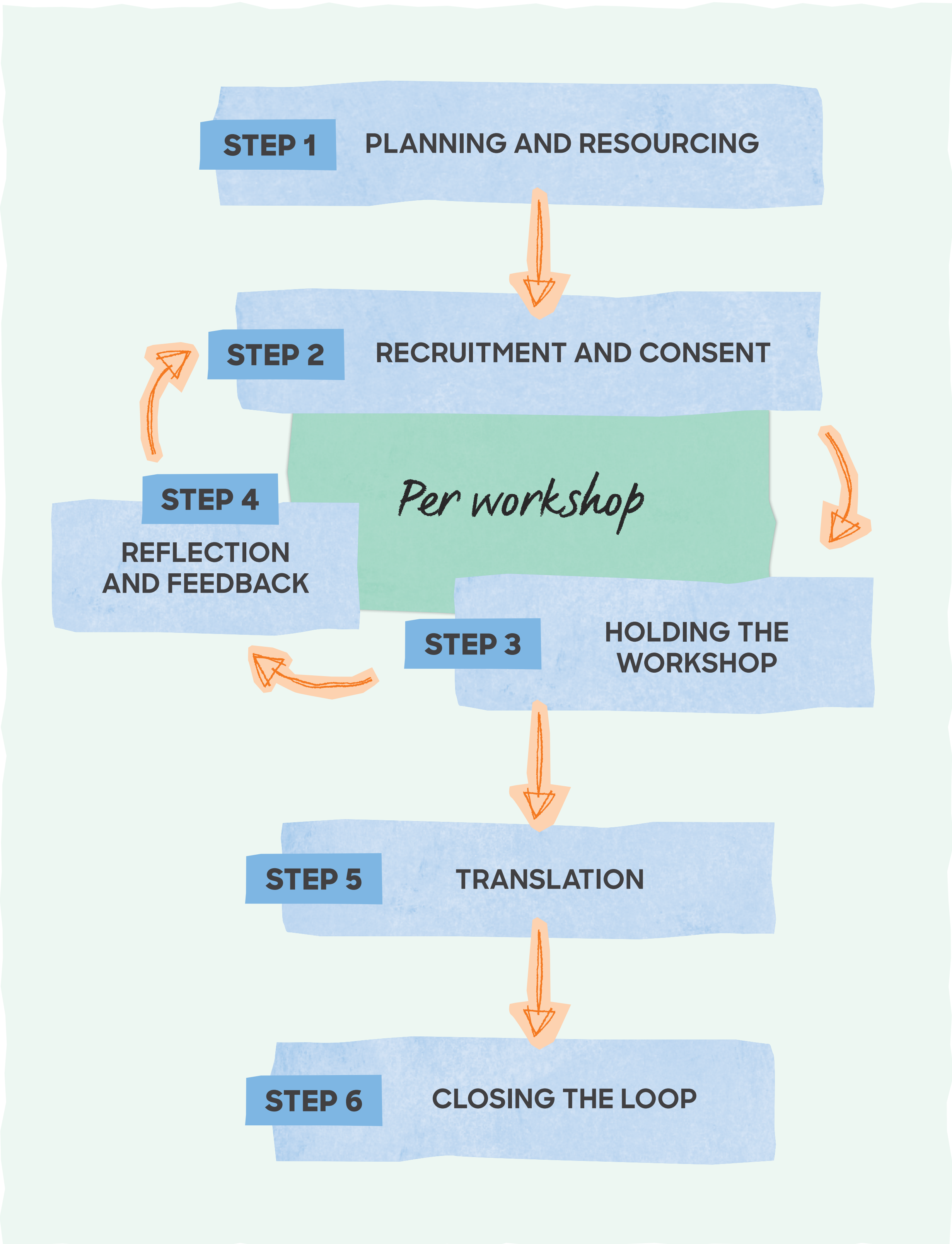

STEP 1

Planning and resourcing

Designing your project

The #chatsafe Model can be used across many settings, fields and topics. When designing your project, it is important to consider:

- What does your project hope to achieve in the short- and long-term?
- What is the creative output being designed?
- Who is eligible to attend your workshop? Consider age range, demographics, and any relevant lived experiences.
- What types of input from young people, or other stakeholders, will you need to achieve your aims? How many participants and how many workshops might be needed as a result?
- Are there opportunities to step up the participatory ladder at all? See page 6.
- Do you have sufficient resources (funding, staffing etc.) for this level of involvement? If not, you might need to step down the participatory ladder.
- Do you have processes set up to appropriately create a safe environment, and, if relevant, support participants with lived experience of mental ill-health, suicide, or any other relevant life experiences? See guidelines by Webb et al., 2023.

Timeline

Like most participatory approaches, the #chatsafe Model takes a lot of time and planning. Sometimes this might be more time than you expect -- especially if you are working in an unfamiliar location, with new stakeholders or with a marginalised community. If you are working in a research framework and plan to publish your workshop findings, you will also need approval from a Human Research Ethics Committee.

Throughout this toolkit, we have given some timeline suggestions for different project steps.

Tip

It can take months to plan workshops, especially if you are going through research ethics approvals. Now that we have approvals and are quite experienced with planning, we start organising individual workshops at least **4-5 weeks in advance** by confirming date, venue, collaborators, and facilitating staff.

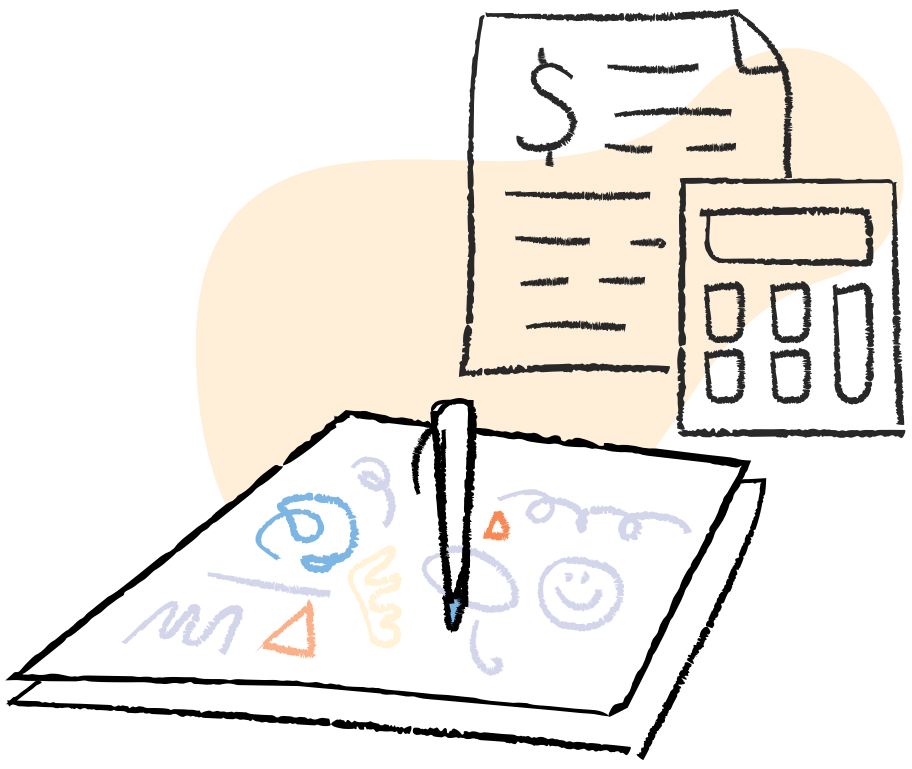

Budget

Remember to budget for:

- staff time
- staff travel requirements (e.g. flights, accommodation)
- room hire costs
- catering
- physical resources (see page 9)
- participant honoraria or reimbursements (aligned with any paid participation frameworks or policies relevant to your organisation) and/or participant travel expenses
- any additional evaluation costs (e.g. cost of survey software, or additional participant payments for completing evaluation activities).

Tip

We pay participants through direct bank transfer after a workshop, but in some contexts it may be more practical or appropriate to pay in cash or with vouchers, which are ideally gift cards that can be used at multiple locations.

Workshop timing

This workshop structure is flexible in its schedule, delivery and timing -- as is the nature of youth engagement. In our experience, a single workshop following this structure has taken approximately 6 hours in total, if delivered in one day. Workshops can also be split across multiple days, if desired.

Regardless of the timeframes given, all workshops should include three components of the workshop (see page 19).

The number of workshops will vary depending on the goals of your workshops.

Physical resources

We recommend bringing:

- stationery (e.g. name tags, sticky notes, pens/pencils/markers, butcher's paper),
- craft supplies (e.g. stickers, cardstock, scissors, glue)
- fidget/sensory toys (e.g. play dough, fidget spinners, stress balls)
- phone stands and a USB-C or Bluetooth microphone (if participants will be using their phones for recording audio or videos)
- snacks and a water station
- hard copies of any resources/ information relevant to the workshop topic, that participants might need to refer to during the workshop
- voice recorders (although you won't need to record the full workshop, you may wish to record some sections like group discussions).

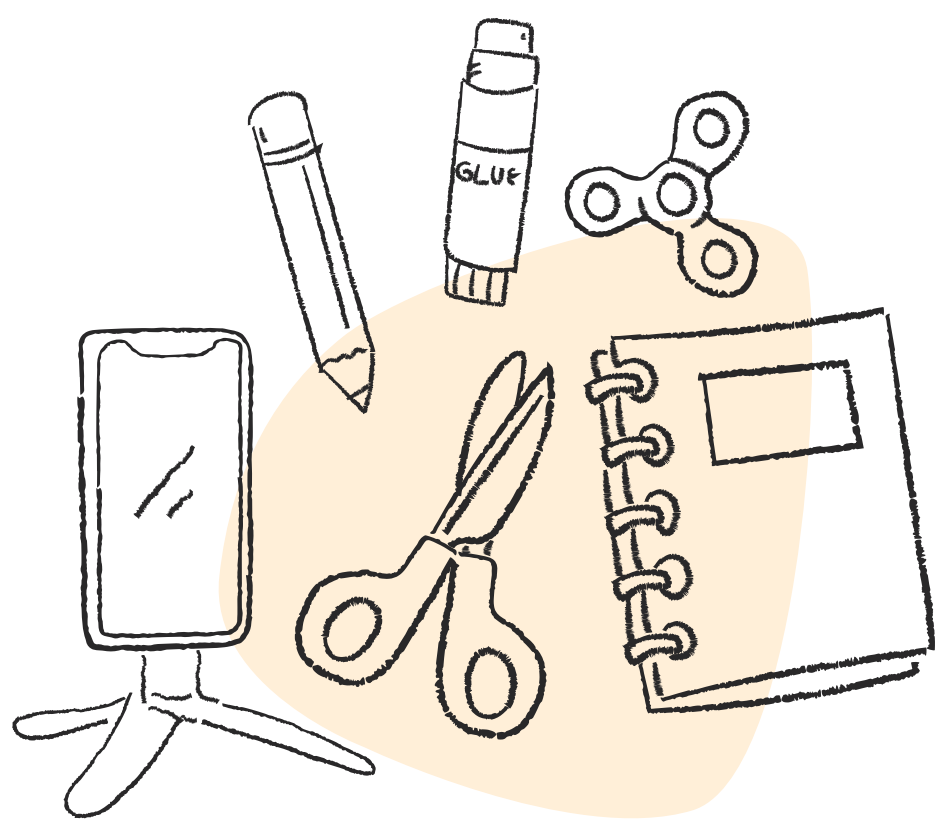

Location and room set-up

These workshops are designed to be delivered **in-person**.

Ideally, **two rooms** will be booked: one with AV equipment and enough seats, tables and food, and another smaller room that can serve as a breakout or quiet space, with additional sensory toys and craft supplies.

We recommend setting up the main room with small tables of 4-6 young people each, with sensory toys, sticky notes, butchers paper, and other relevant resources in the middle of each table.

Where possible, ensure the space is accessible for all participants, including those who use mobility aids such as wheelchairs. Venues should also be close to public transport.

Tip

If participants will be capturing photos or video in a shared space (like a library or a community hub), remind them to be respectful of other people and make sure others are not in the background.

Tip

If only one room can be booked due to resourcing or other constraints, think of other locations that participants might use to take a break -- like a lounge, café, or an outdoor area such as a courtyard. Make this option known to participants during the workshop.

Suggested room set-up

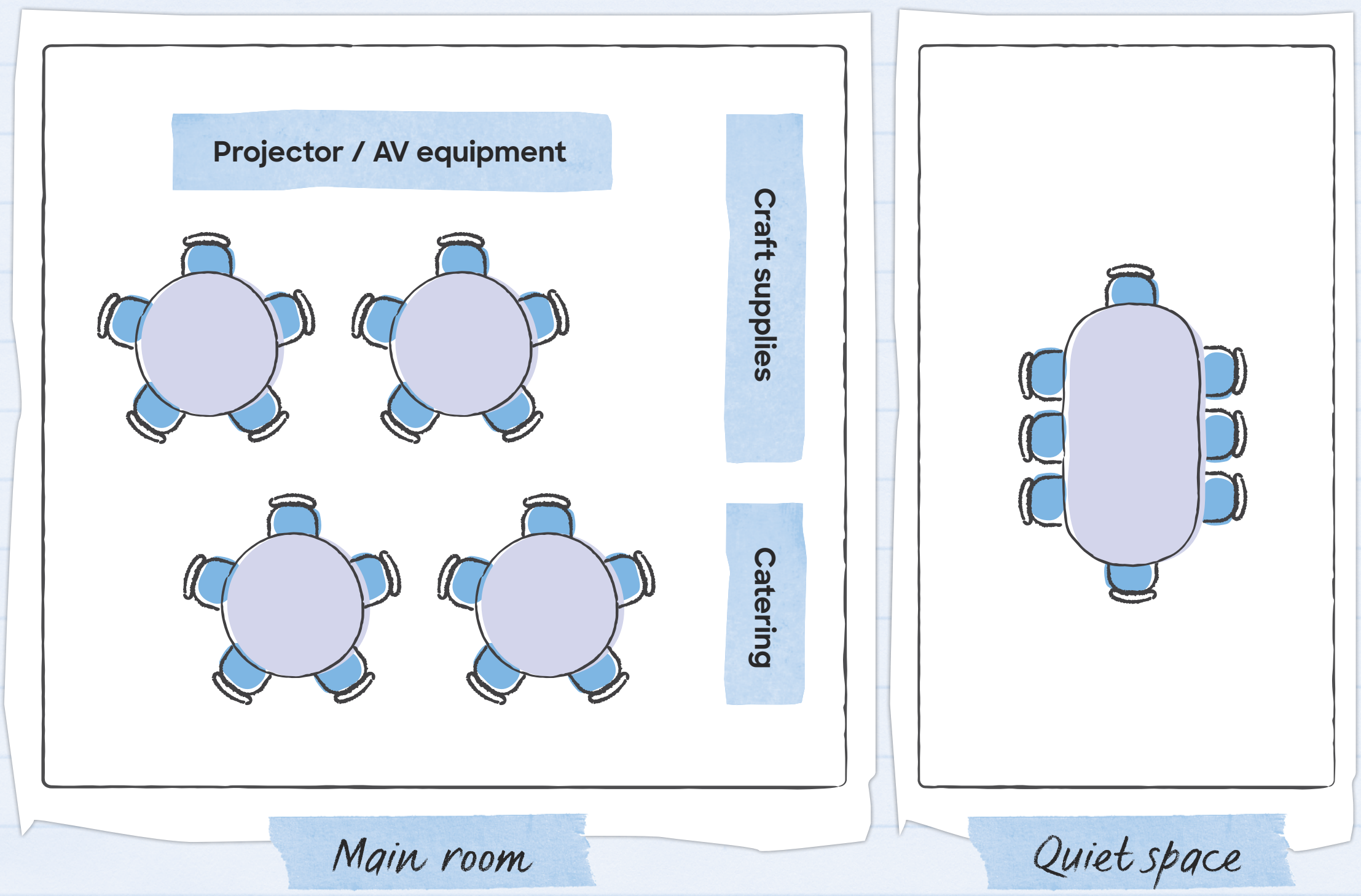

Staffing and roles

There are no strict rules for how many staff should facilitate a workshop. The main consideration is ensuring the ratio of staff feels proportionate to the number of young people in the room -- it should not feel 'staff-heavy'. In our experience, three staff are sufficient for 8-10 participants, four for 11-20.

Ideally, facilitators will be familiar with the topic and trained in leading workshops, focus groups, or similar activities (e.g. qualitative researchers, social media experts). Depending on the topic of workshop, they may require additional training, such as Mental Health First Aid training.

If the workshop involves working with young people under the age of 18 years, all staff should have appropriate background checks for working with minors in your jurisdiction (in Australia, this includes a Working with Children Check).

Roles include:

Main facilitator: This person will be in charge of clearly explaining the workshop activities and instructions. This will involve standing at the front of the room, presenting, and ensuring that the young people understand the instructions.

Co-facilitator: This person will play a key role in supporting the main facilitator with activities, monitoring participant wellbeing, and keeping engagement among participants. Tasks might involve keeping time, controlling slides, and monitoring for signs of distress. They also may lead some activities.

Field note-taker: This role involves observing participants, writing down reactions and discussion content, and capturing feedback. This person may use a voice recorder to record some, or all, of the group discussions. They may also provide practical support in other ways, such as being the contact person for catering delivery.

Further facilitator recruitment advice:

- It is a great idea for at least one facilitator to also be a subject matter expert, such as a communications professional, if you are designing a social media campaign, or a clinician if you are developing a mental health intervention.
- If resourcing allows, we encourage you to involve a young person in a facilitation role. It is important to provide adequate training and support to any youth facilitators in advance of the workshop.
- An identified mental health professional should be available to provide support for participants if needed. This person may be available either in person or be on-call, depending on the context of your workshop. See **Safety Management Strategy, page 16.**
- If working with a partner organisation, it can be valuable to also have a partner representative in attendance for part of the day, or the whole day. This person may provide practical support, but can also help build trust with participants who may already have rapport with this person.

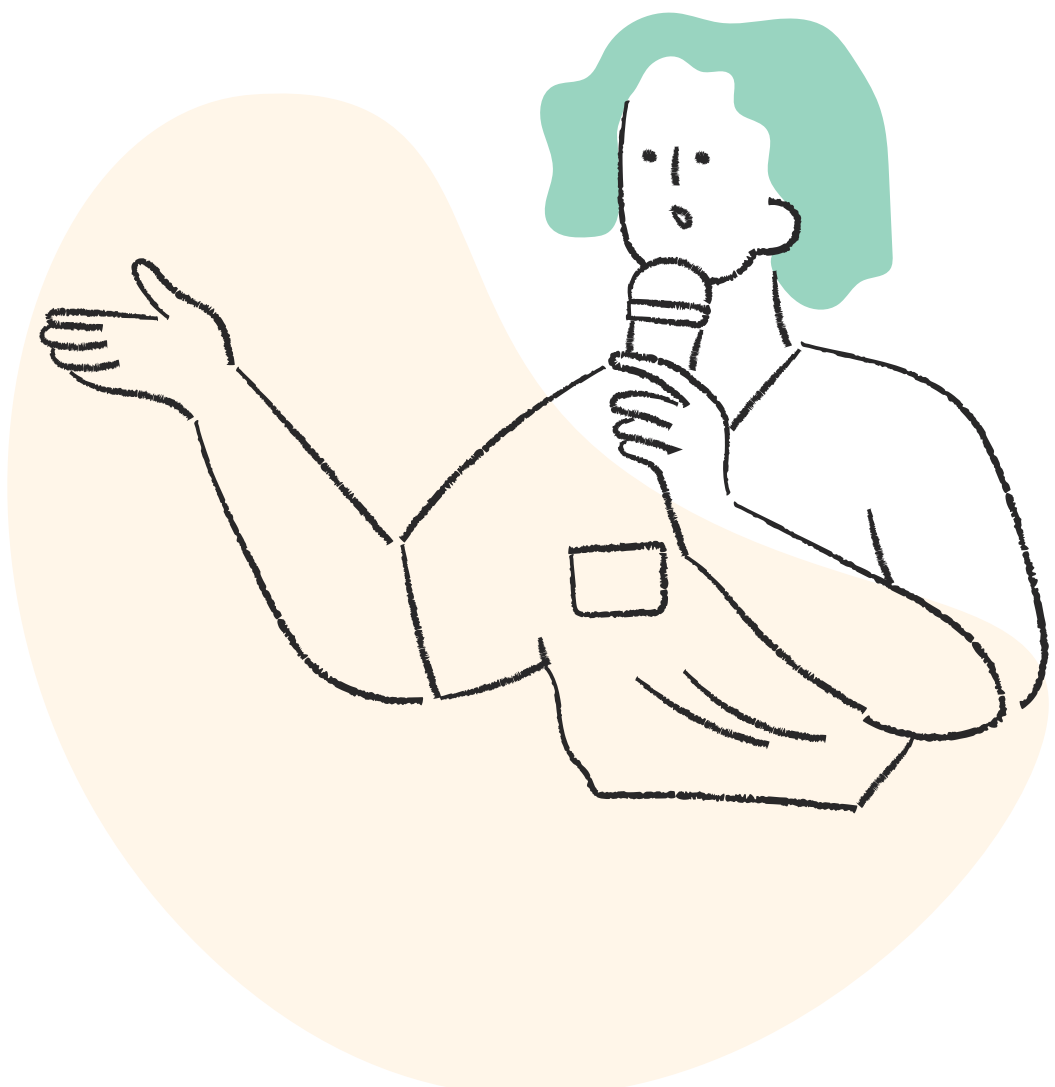

Evaluation planning

Evaluation involves generating evidence to understand the extent to which a program or activity is meeting its objectives, and to understand its impact. Evaluation can result in improvements to the design, implementation, and outcomes of a workshop.

Workshop evaluation activities can be incorporated within the #chatsafe Model, though formal evaluation may not be needed in all contexts.

Evaluation planning should cover the following:

- What do you hope to achieve with your workshops?
- How might you evaluate their **acceptability**, **feasibility**, and **impact**? What indicators and evaluation tools might allow you to measure these?
- What types of data will you collect before, during, or after workshops? How might you collect data? e.g. hard copy feedback forms, online registration forms/follow up surveys, interviews. On how many occasions?
- How intensive and time consuming will any data collection be for participants?
- How might you manage, store, and analyse this data?
- What are the relevant ethical considerations? Will you need approval from a Human Research Ethics Committee in order to collect and report data?
- How will you synthesise and report results?

When designing your evaluation, we recommend using the Youth Engagement Evaluation Framework (Aston & Tse, 2022) as a guide (see Resources, page 34).

Tip

Keep any survey materials brief and user-friendly to improve engagement. For fields that are not essential, consider including a 'prefer not to say' option.

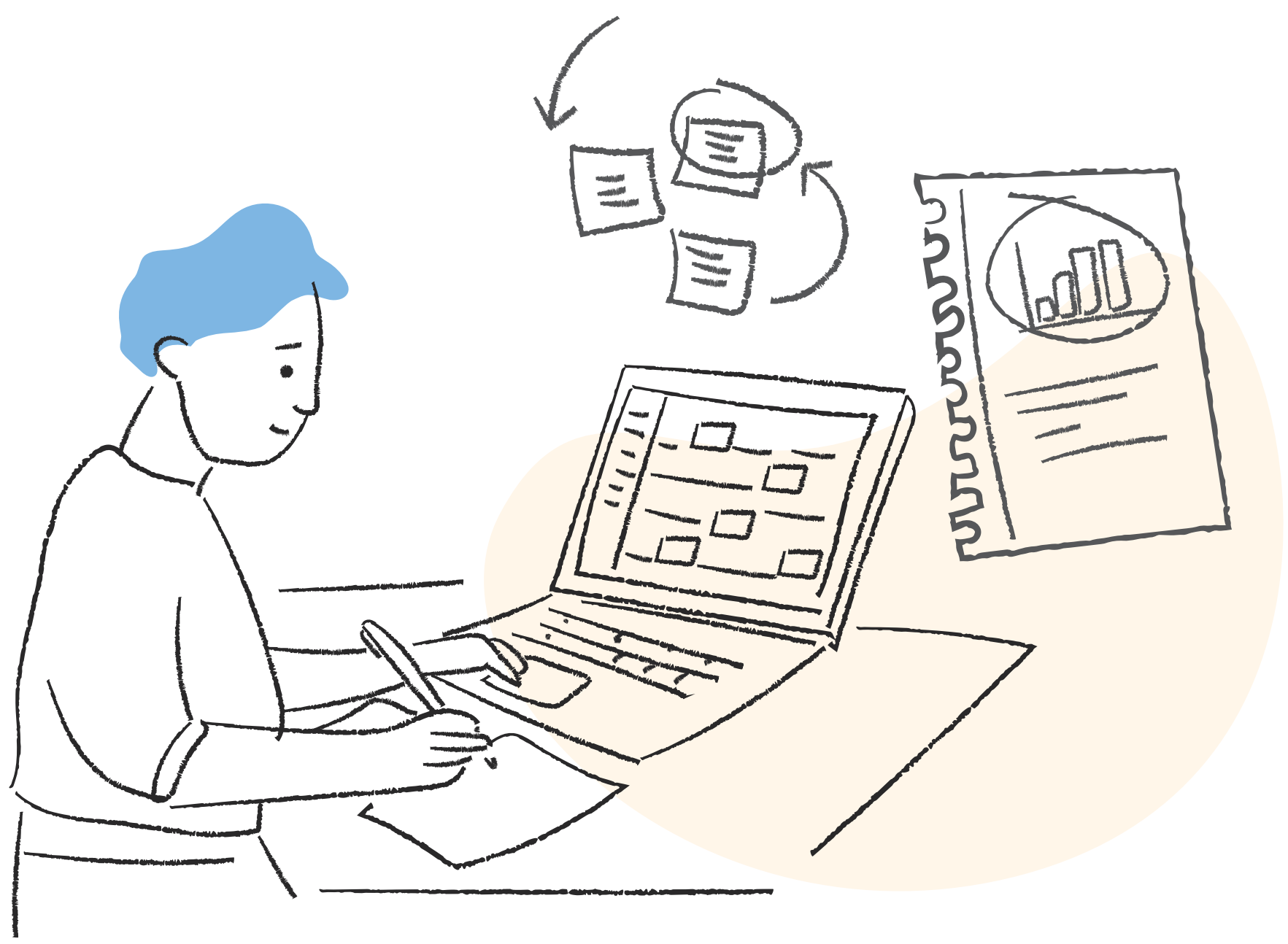

STEP 2

Recruitment and consent

Advertising: 3-4 weeks before the workshop

The #chatsafe Model involves advertising the workshops primarily online. Recruitment can occur in many ways. Your organisation may already have access to a network of interested young people, or you may need to recruit more widely.

Recruitment can occur through existing networks and contacts established within your sector. You may wish to reach out to a potential collaborator to assist with recruitment. Contacts from collaborating organisations can be emailed recruitment information or given hard copy flyers, posters, or advertisements to circulate to young people in their networks.

Young people can also be encouraged to share recruitment flyers with their peers – a process known as 'snowballing'.

Social media advertising can be used, including organic posts (those posted on your organisation's social media accounts) and paid advertising.

Any recruitment materials should direct participants on how to register for a workshop, including information about:

- the purpose of the workshop
- what to expect
- location
- date and time
- a link to a registration form.

The #chatsafe Model is designed for up to 30 participants maximum, but typically 12-15 participants are ideal. If you're anticipating a large number of signups, it can be useful to have an expression of interest form (rather than direct registration) and automatically close sign-ups after a maximum number has been reached.

Checklist:

- Create recruitment strategy
- Determine inclusion and exclusion criteria
- Budget for any paid advertising
- Create recruitment flyers or ads
- Create online registration form

"I was recommended to bring a friend… I think that was a really good thing because I could like send out the invite to people who usually wouldn't come to workshops."

workshop participant

Registration process

Here is an example of the full registration process, with all components being built into one form e.g. a Google Form. Information about each stage is included in this toolkit.

Tip

- Eligibility screening questions can ask about demographic details such as age and gender, and any other characteristics that are relevant.
- In our experience, registration works best when it is as short and simple as possible – only one link, registration occurs in one step, and the language in each form is clear and user friendly.
- Ensure you collect participant contact details, and ideally their preferred method of communication e.g. SMS, phone call, email.
- If you are paying people via bank transfer, you can collect their bank details at the time of registration, or request this afterwards.
- It's a great idea to get a young person to test your registration form before you send it out.

Suggested illustration

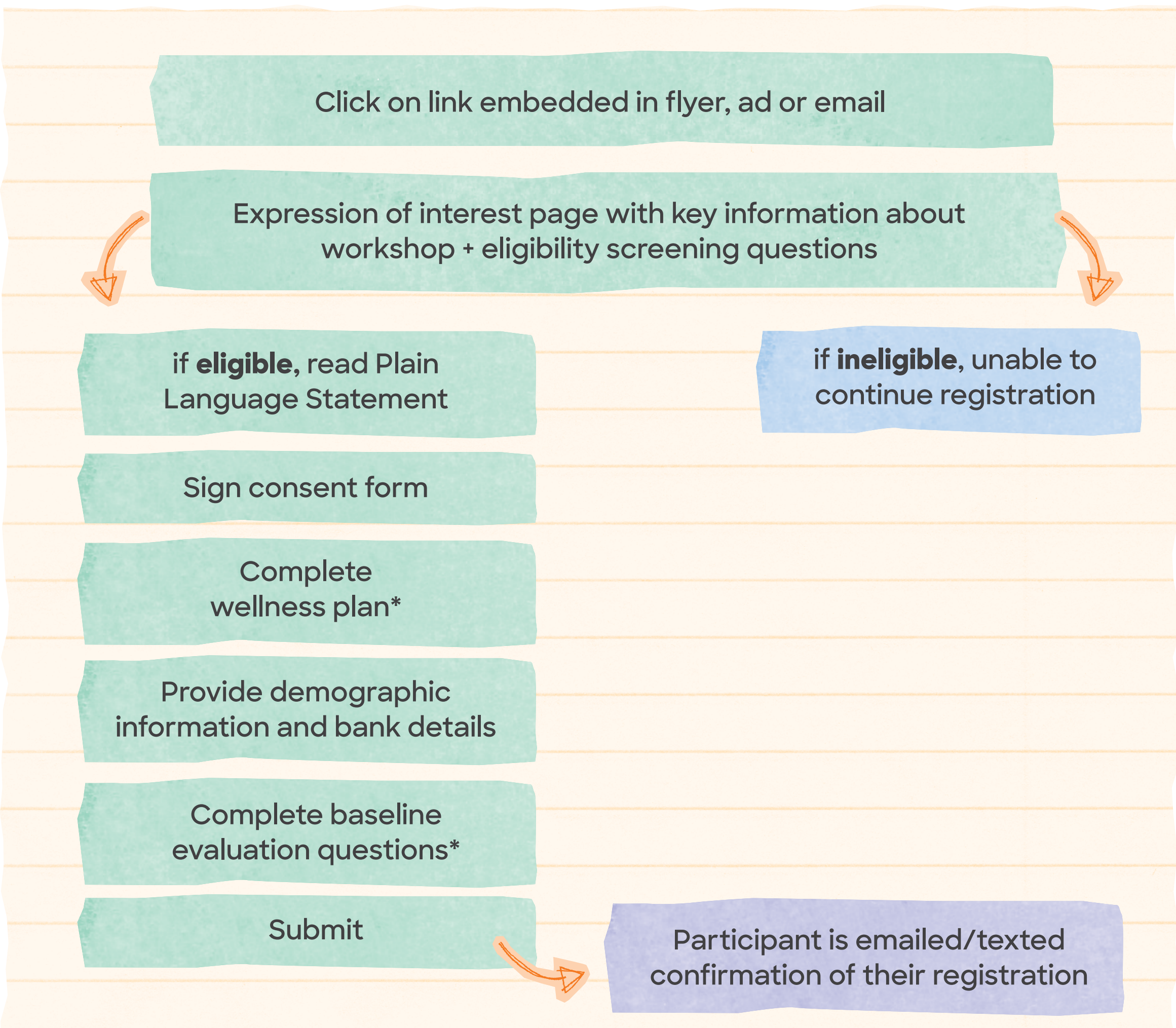

Items marked with an *asterisk may not be relevant in all contexts.

Informed consent

It is essential that young people understand what they are agreeing to when they register to attend a workshop. They should know:

- what to expect from the workshop
- how their ideas will be used
- what payment is involved, and minimum involvement to receive this payment e.g. attending the full day
- if recording will occur
- how you will protect their privacy (such as not including identifying details in any reporting)
- what supports are available should they experience distress
- that they can withdraw from the workshop at any time.

This is especially important if you are working in a research framework and will be collecting or publishing data. If this is the case, you will need to follow research ethics guidelines relevant to your jurisdiction.

Information should be provided in advance through a plain language summary, and participants should sign a consent form. Ideally this will be part of registration, and can be completed online. However, if it's not possible to complete consent forms in advance, bring forms to the workshop and ensure these are completed at the start.

For participants who are aged under 18, you will need to seek parental consent too. This might be a written consent form or verbal consent.

Participants may need to sign other forms like **media release/assignment of rights/ use of image** forms during the registration process. They may also need to provide information like their bank details, allergies and any other dietary requirements, and accessibility requirements.

Checklist:

- [x] Create plain language statement and consent form, and any other required forms (ideally embedded within your registration form)
- [x] Create similar forms for parents/guardians, if participants are under 18 years

Tip

- A short video consent form can help communicate key information in an accessible way. Using graphics, photos and other visuals can also help break up information and keep it engaging.
- Sometimes, young people need to seek consent from a parent or guardian who does not speak the language that the forms are written in. To help this process, consider working with a translator or trusted contact in the community who can explain the workshops in the same language, and obtain verbal consent.

Safety management strategy & wellness plans

The #chatsafe Model was originally designed for youth participation activities relating to suicide prevention. Therefore, safety and wellbeing are important considerations.

If the workshops involve sensitive topics, a safety management protocol – including collection of wellness plans – is integral.

Tip

Using the #chatsafe model with young people who have lived or living experience of suicide?

Check out the **Guidelines for involving young people with lived and living experience of suicide in suicide research** by Webb et al. (2023) – see Resources.

Safety management might involve some, or all, of these components:

Before a workshop, during registration

- Ask participants to complete a wellness plan (more info on page 17).
- Provide relevant helpline and support information. For example, the numbers of reputable 24/7 mental health or suicide prevention helplines, including any that might be particularly relevant to the target audience of your workshop -- such as LGBTIQA+ helplines.
- Inform young people about how they will be supported during a workshop if they are distressed.
- Inform participants that they can withdraw at any time.
- For workshops in the context of mental ill-health or suicide prevention: facilitators should determine procedures for responding if a participant discloses current suicidal thoughts, intent or plans, including how to escalate concerns to a mental health professional or emergency services should there be immediate safety concerns.

During a workshop

- Remind participants of helpline and support information in presentation slides, handouts, or other materials.
- Remind participants to take breaks and look after themselves.
- Participants should be informed who they can speak to, such as a co-facilitator, if they are distressed or need to take a break.
- Discuss and set room agreements with the participants.
- Monitor participants for signs of distress. A co-facilitator should hold onto wellness plans and support participants if they need to speak to someone.

After a workshop

- When thanking participants via email, text or phone, provide helpline and support information once more should they need support after the workshop.
- Consider your duty of care, and if any proactive follow up might be required for any participants who withdrew and/or experienced distress during the workshop.

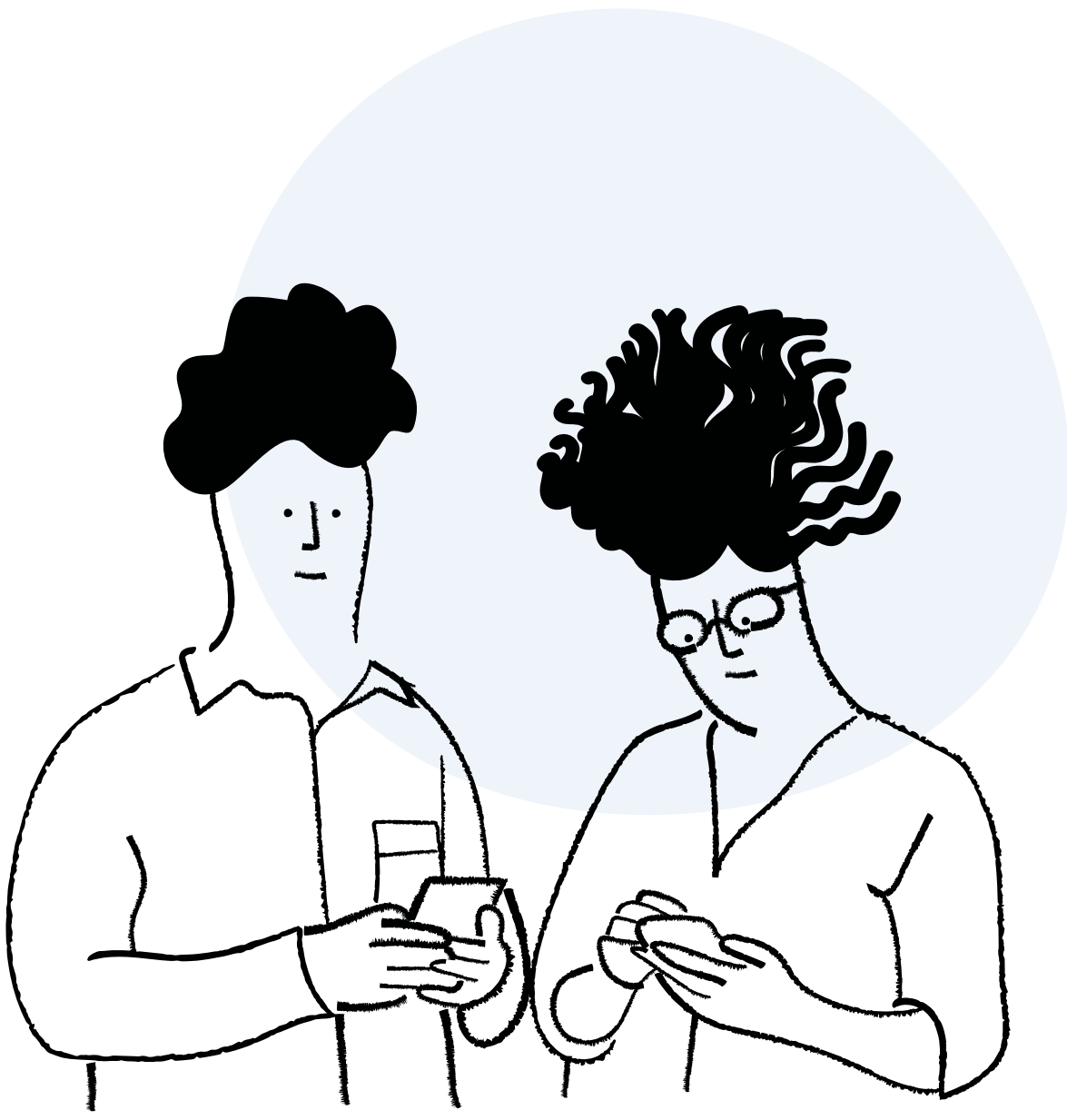

Wellness plan template

Wellness plans are recommended for workshops on the topic of suicide prevention or other topics that could be distressing for participants.

Wellness plan prompts may be embedded in registration form, or they might be circulated in another way, such as a hard copy (like the template below), to be completed at the start of a workshop and handed to the facilitators.

While some fields may be presented as compulsory, such as emergency contacts and summarising any relevant physical/medical conditions or allergies, some of these fields may be optional.

Young people we have spoken to have reported that they are comfortable completing wellness plans – especially if some fields are optional, they understand **why** this information is collected, the information is clearly **relevant** to the topic of the workshop, and they trust that the information will be **stored privately and used appropriately**.

"I just feel like the Wellness Plan is kind of necessary. So I didn't really have any problems with it."

workshop participant

"I think it's good, especially because it's related to the topic and like mental health and suicide... it's really good to ask these questions because definitely we will come from different lived experiences."

workshop participant

Suggested wellness plan prompts (please see Appendix for a downloadable template)

Emergency contact (this should be a parent, guardian or another responsible adult):

- Name:
- Relationship to you:
- Mobile number:

Professional support person e.g. doctor, psychologist

- Name:
- Relationship to you:
- Phone number:

Health and wellbeing:

- Any physical/medical conditions or restrictions? e.g. food allergies, asthma, epilepsy etc.
- Any mental health information we may need to be aware of?
- Some signs or symptoms that you notice when you are becoming uncomfortable or distressed:
  - Are there any situations, activities or topics that may make you feel uncomfortable, distressed, or may trigger these symptoms?
  - What sort of activities, tools, or resources you use to help you relax? e.g. spending time with friends, listening to music, yoga etc.
  - Things you have done in the past that have helped when you were feeling unwell e.g. talking to a friend or a family member, using a helpline, or using strategies suggested by your psychologist.

STEP 3

Holding the workshop

Pre-briefing and finalising: up to 1 week before the workshop

A pre-brief is a planning meeting held a few days in advance of a workshop. It is an opportunity to touch base before a workshop, to ensure all facilitators are on the same page.

All facilitators should attend, plus any other staff who are supporting the workshop (such as those with administrative responsibilities) or who are key stakeholders.

This meeting should cover:

- Which staff are attending on the day and their roles and responsibilities.
- This includes who will greet participants, who will run each activity, who will take notes/look after audio recording, and who will provide wellbeing support if needed.
- Time, date, and venue of workshop, plus any other logistics.
- How many participants have registered for the day, their demographics, and anything to note from their wellness plans, such as if participants have flagged how best to support them on the day.
- Any admin required prior to the workshop day, such as following up any missing parental consent forms.
- Any adjustments required to the workshop activities based on the demographics of participants who will be attending, such as modifying content for younger participants.
- How staff will communicate on the day e.g. a group text chat.

Tip

We have a standing agenda template for our pre-brief meeting (and our debrief meeting), which we adjust based on the context of each workshop - see case study on page 28.

Checklist:

- Create recruitment strategy
- Hold pre-brief
- Ensure all registered participants have completed required paperwork
- Send email & text reminders to participants
- Confirm snacks and catering
- Confirm all roles and responsibilities
- Confirm your agenda for the workshop
- Pack up all your materials
- Print agendas, wellness plans, a participant list, and spare consent forms

Workshop structure

After an introduction activity, a #chatsafe Model workshop comprises three key components:

1. Learn and Define

This is the first component of the workshop and focuses on understanding, connecting, and learning.

The context is then defined to ensure all participants are on the same page. This usually involves a presentation or talk about the topic, and overviewing relevant resources or evidence.

Workshop content during Learn and Define can be specific and bespoke to the experiences and concerns of the young people in the room.

2. Design

In this component, young people and facilitators will conceptualise, collaborate, and create in response to a design brief.

This component will take up more time than the other components of the day.

This involves a brainstorming activity, followed by time to create and refine one or more ideas, individually or in small groups.

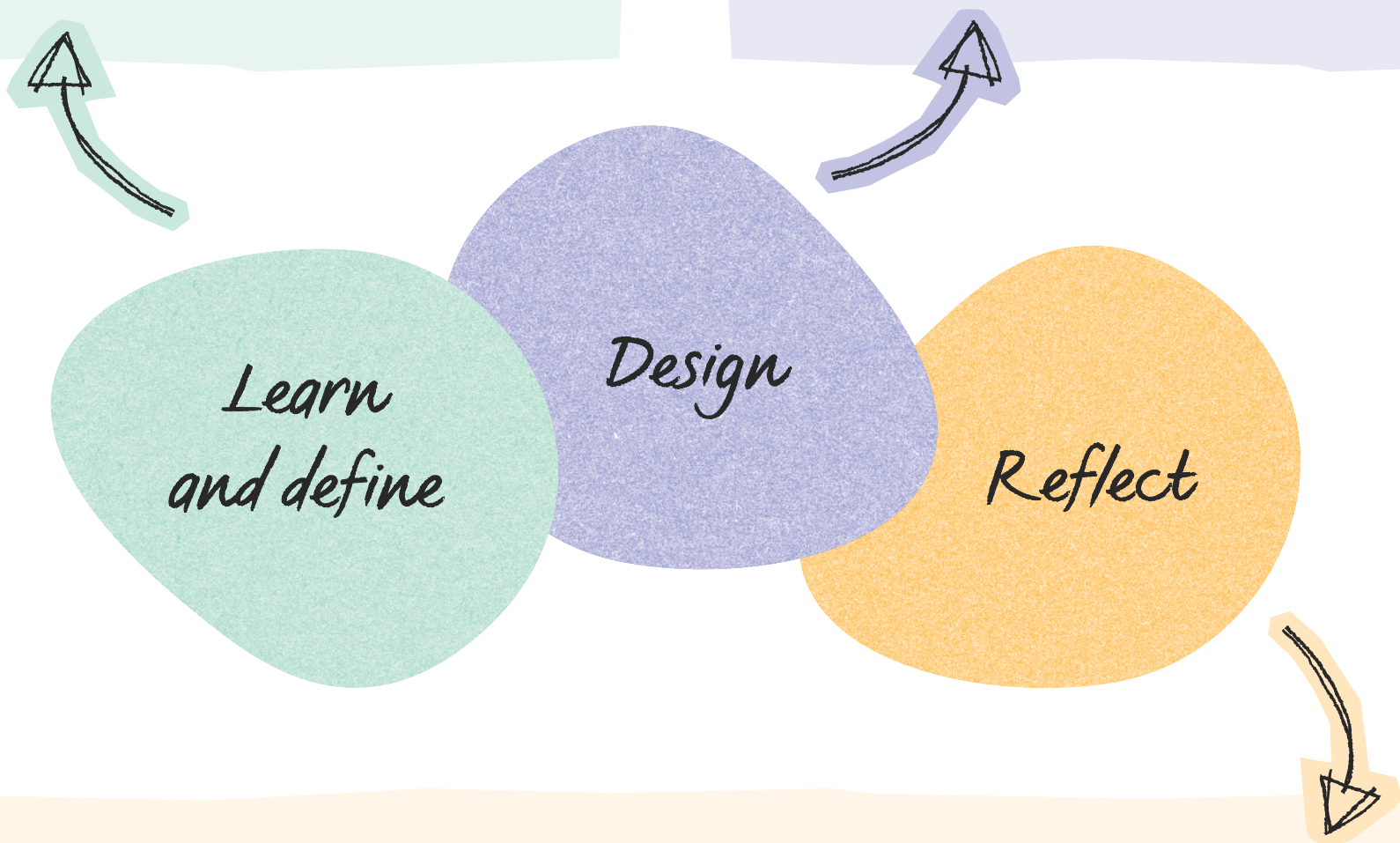

3. Reflect

During this component, young people will share their designs and creations with everyone present.

Young people and facilitators will also engage in discussion regarding the experiences of the design process. This will involve the sharing of knowledge and learnings experienced throughout, and expanding on ideas that were discussed throughout the day.

This may also involve reviewing challenges and benefits young people experienced as part of their participation, and capturing what facilitators and barriers young people perceived during the workshop.

Researchers here will facilitate discussion to ensure all participants have opportunity to present and reflect.

Workshop agenda example

Time	Section	Content
**8:45am**	Facilitators arrive	Facilitators arrive to set up tables, presentation slides, stationery, sensory toys, craft supplies etc.
**9:45-10am**	Participant arrival window	Facilitators greet participants, tick their name off the roll, and ask participants to create name tags, including their pronouns.
**10am**	**Introduction**	• Main facilitator welcomes participants and overviews what to expect during the workshop.<br>• Housekeeping e.g. location of bathrooms, the quiet space.<br>• Ice-breaker activity.<br>• Room agreements -- set expectations around the workshop, discuss group guidelines and wellbeing support.
**10:30-11am**	**Learn & Define**	Main facilitator overviews the workshop topic, defining the problem.
**11-11:15am**	Break	Snacks and water provided.
**11:15-12:30pm**	**Design**	Brainstorming activity.
**12:30-1:15pm**	Lunch break	Lunch provided.
**1:15-3pm**	**Design (continued)**	Design activity/prototype creation.
**3-4pm**	**Reflect**	Participants present ideas created.<br>Reflection activity.<br>Cool down activity.
**4pm**	Workshop ends	Pack-down.

Activity & discussion ideas

Introduction

- Facilitators welcome participants to the space and introduce themselves.
- Facilitators remind participants about the purpose of the workshop.
- Housekeeping e.g. location of toilets, location of quiet space.
- Ice-breaker activity.

> *"[The group rules discussion] kind of set a tone for discussion… I think it really opened up that we're all friendly. We're all here to talk and we'll be all respectful to each other."*
>
> **workshop participant**

Ice breaker ideas

- **Ball game**
  Participants stand in a circle. One person holds a ball (or a soft toy), and while they do, they say their name, pronouns and answers a prompt e.g. 'What is your dream job? If you could live a day in the life of anyone past or present, who would it be?'. The participant throws the ball to another person, who does the same, until everyone has introduced themselves.
- **Whose story?**
  Everyone writes one silly/funny story that has happened to them on a scrap piece of paper and places into a hat/bucket/ pile in the middle. One by one, participants pick one out and read it out, the group tries to guess who the story belongs to.
- **Boring fact**
  Participants walk around in a circle until the facilitator says 'stop'. Participants then turn to the person closest to them and they play a quick round of rock, paper, scissors. The winner of the round must then tell the most boring possible fact about themselves to their partner e.g. 'I put my socks on before my shoes', 'I have soy milk in my coffee'. Facilitator then says 'start' and participants begin walking around the room again, repeating the process with a different partner.
- **String game**
  Participants pass around a ball of string, and when holding the string mention a fact about themselves. A participant who relates to the fact then takes the string, and mentions a new fact. This continues until everyone has spoken. The string will become tangled and interwoven.

Setting expectations, group guidelines & wellbeing

- Remind participants of reasonable outcomes from a one-day workshop, and they are not expected to be expert in the topic or to create polished ideas.
- Remind participants about the scope of the workshop. For example, that you might not be able to use all of their ideas in the final product.
- Room agreements are then discussed as a group:
  - Prompt: 'What are some good group rules we can all agree to, so that we meet our goals today?'
  - Facilitator to write these on butchers' paper or on a white board.
  - Facilitators can prompt participants if key topics don't get mentioned organically. For example, respecting one another, being mindful of the language they use, and thinking about what they are comfortable sharing.
  - If feasible, ask participants to initial or sign the board as part of the room agreement.
- Remind participants to think about what they wrote in their wellness plan (if a wellness plan was created prior to the workshop) and consider: 'What I comfortable/ uncomfortable sharing today?', 'What can I do if I feel uncomfortable today?'
  - Inform participants who to speak to if they need support e.g. speak with or text a co-facilitator, and overview suggested helplines.

Room agreement examples:

Call out if you are stuck.

Stay open-minded.

Respect different opinions.

Think about the language you use.

Be considerate of others.

Respect personal boundaries.

Think about what you are comfortable sharing.

Get some fresh air during the breaks.

No idea is a bad idea.

Don't hog, bog, or frog:

- **Hog:** Don't dominate the conversation. Make space for the quieter voices.
- **Bog:** Don't get stuck on one point. Keep things moving.
- **Frog:** Don't jump around too much. Stay with the topic long enough to explore it properly.

Respect each other's' privacy.

There are no silly questions.

1. Learn & Define

- Main facilitator defines the topic/context for young people, to ensure they are all on the same page and have foundational knowledge about the topic/context.
- Involves a brief educational presentation, with or without presentation slides.
  - Give participants plenty of opportunity to ask questions and share their own views.

See our Case Study for more information about what this section looks like for #chatsafe-specific workshops.

Tip

This part of the day is important. By informing and educating participants, you are setting them up for the creative activities later in the day. However, it can feel like a lecture if it runs for too long or is too one-sided.

Aim to keep this part of the day concise, and break up with engaging activities like questions, group discussions and reflection prompts etc.

2. Design

Brainstorming activity

- Inform participants about the design brief, including types of ideas you are seeking (for example, social media content), and any specific prompts (such as the desired format e.g. short form video content).
- Provide examples of what you are seeking, such as ideas created in any previous workshops.
- Ask participants to come up with ideas for something to create today in small groups. Ideas can be written on butchers' paper or sticky notes.

> *"I personally really enjoyed [the creation activity. I had a lot of fun doing it. I really liked working in a small group."*
>
> **workshop participant**

Tip

This activity can be supplemented by discussion or activities to think about existing content/ideas that they find engaging, youth-friendly, or helpful, and what they like about them. For example, you could stick butchers' paper up on the wall and ask participants to respond to prompts to get them thinking.

Tip

If possible, incorporate options for participants to share their thoughts anonymously throughout the day. For example, you could poll participants using real-time survey software e.g. Mentimeter, Slido.

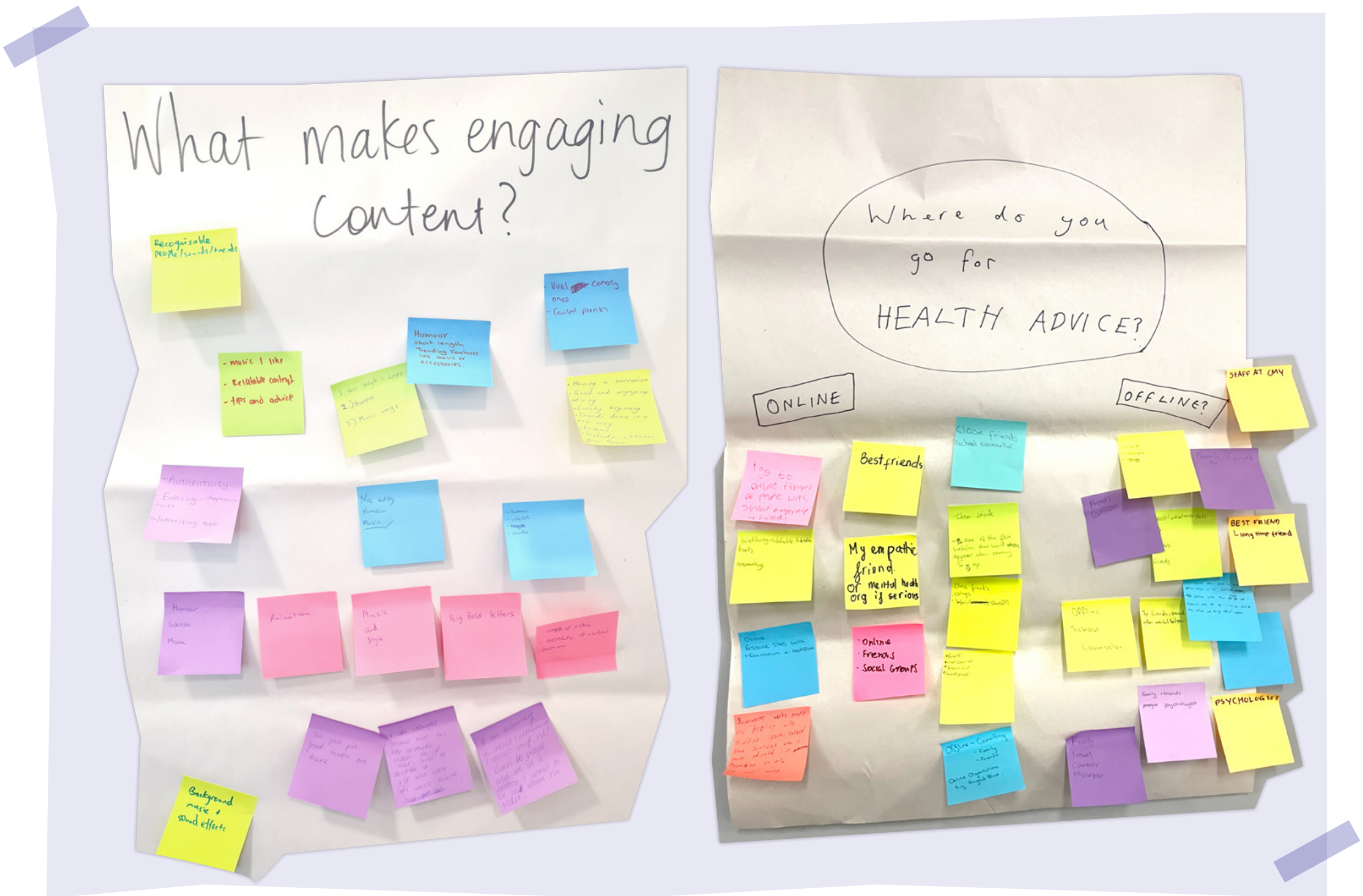

Creation activity

- First, encourage participants to narrow down the ideas they want to work on. From their list of ideas, ask them to select at least one that they are passionate about and can realistically create or prototype by the end of today.
  - Remind participants that you do not expect them to have polished ideas or professional content by the end of the day. Ultimately, you may want something that they can hand over at the end of the day so your team can refine ideas further.
  - Inform participants how the ideas will be shared afterwards e.g. 'After two hours, please AirDrop or email this to us, so we can look at it on the big screen as a group'.
- In groups or individually, participants storyboard, sketch, draw, film, photograph their ideas.
  - Check in with participants to learn about their ideas, answer any questions, and help them if they are stuck.

This can be a largely participant-directed activity.

Many participants have reported this section to be the most fun part of the day.

Tip

- It can be helpful to encourage participants to workshop or prototype multiple ideas – sometimes as many as possible! This helped take the pressure off participants creating one 'perfect' idea, reduced creative blocks, and allowed them to try lots of things to see what might work.
- To avoid participants spending this time working on an inappropriate or unrealistic idea, consider checking in with them at the start of the activity so they can run their ideas past you.
- Avoid crowding participants –– give them time to workshop and refine their ideas, but check in on them occasionally and step in if they need more guidance.

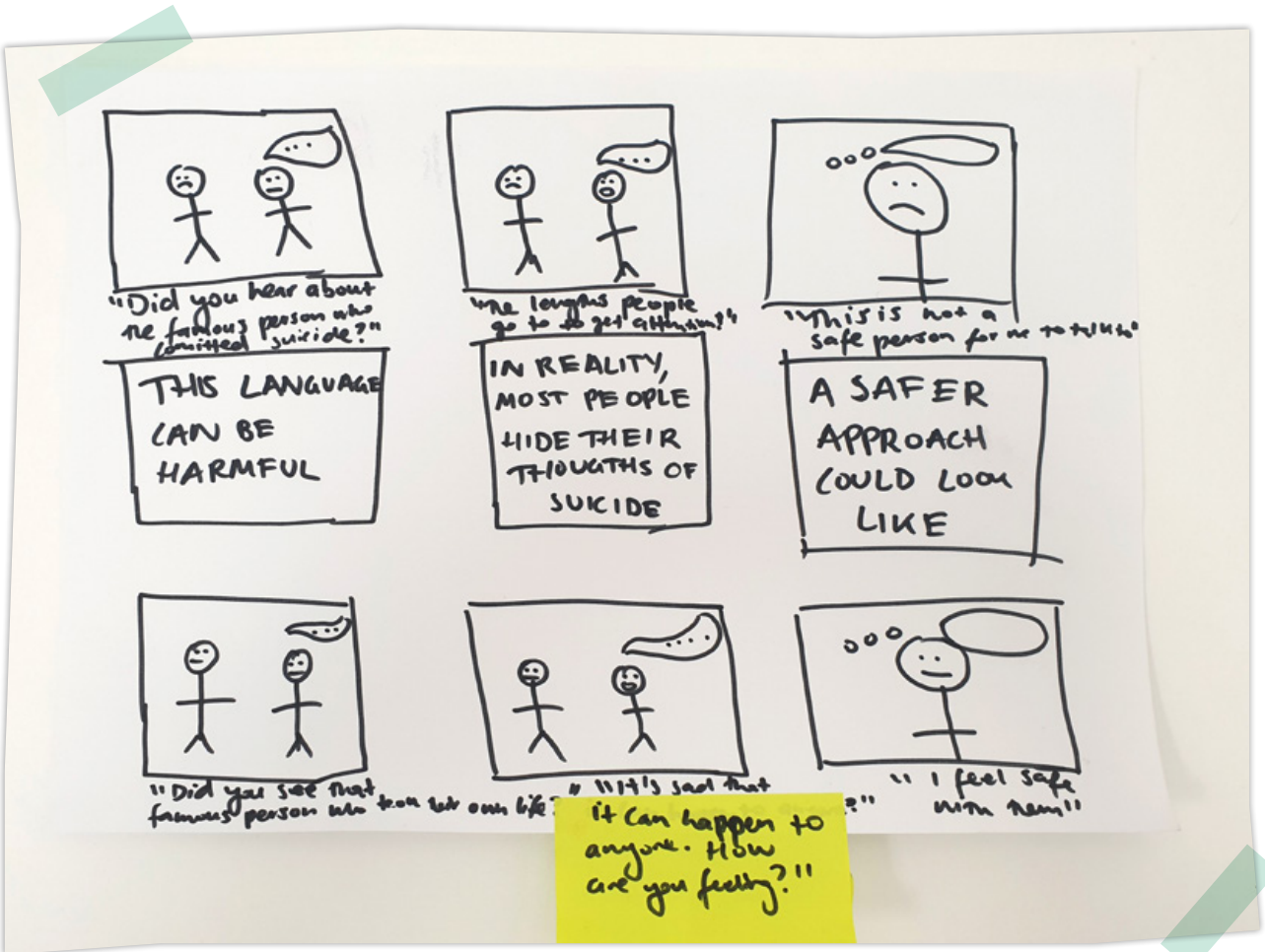

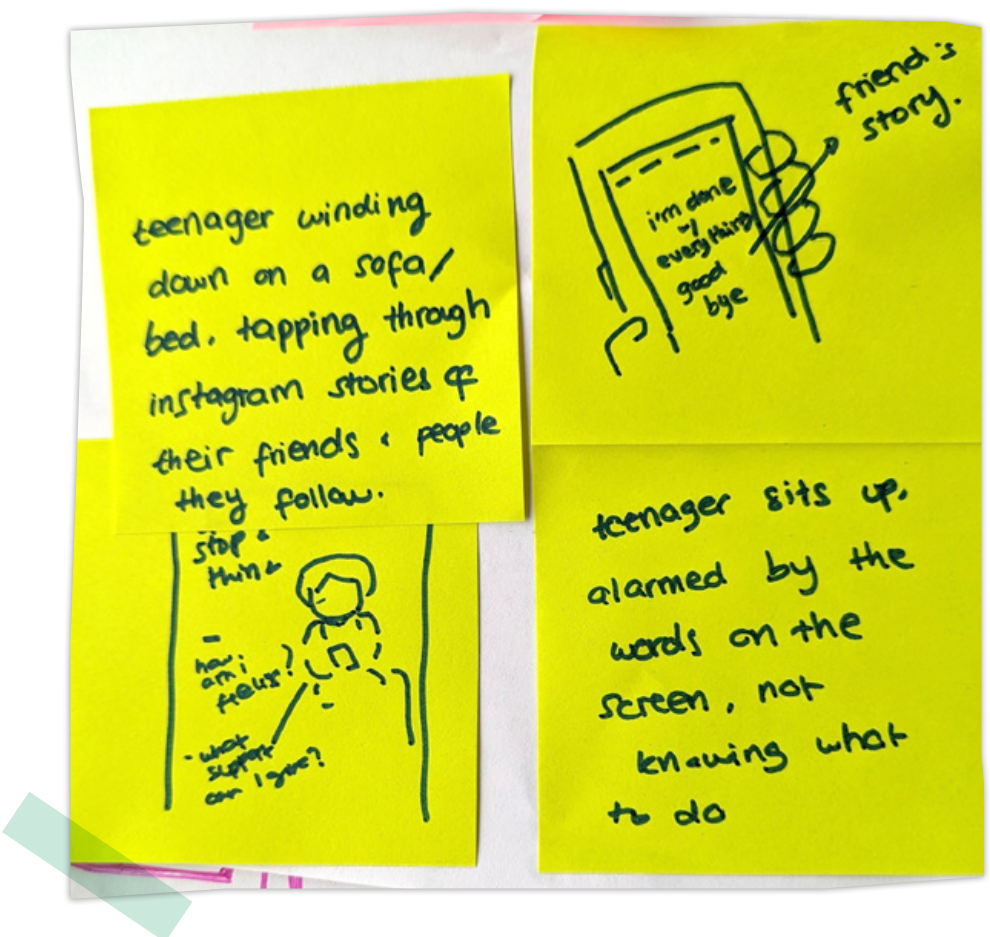

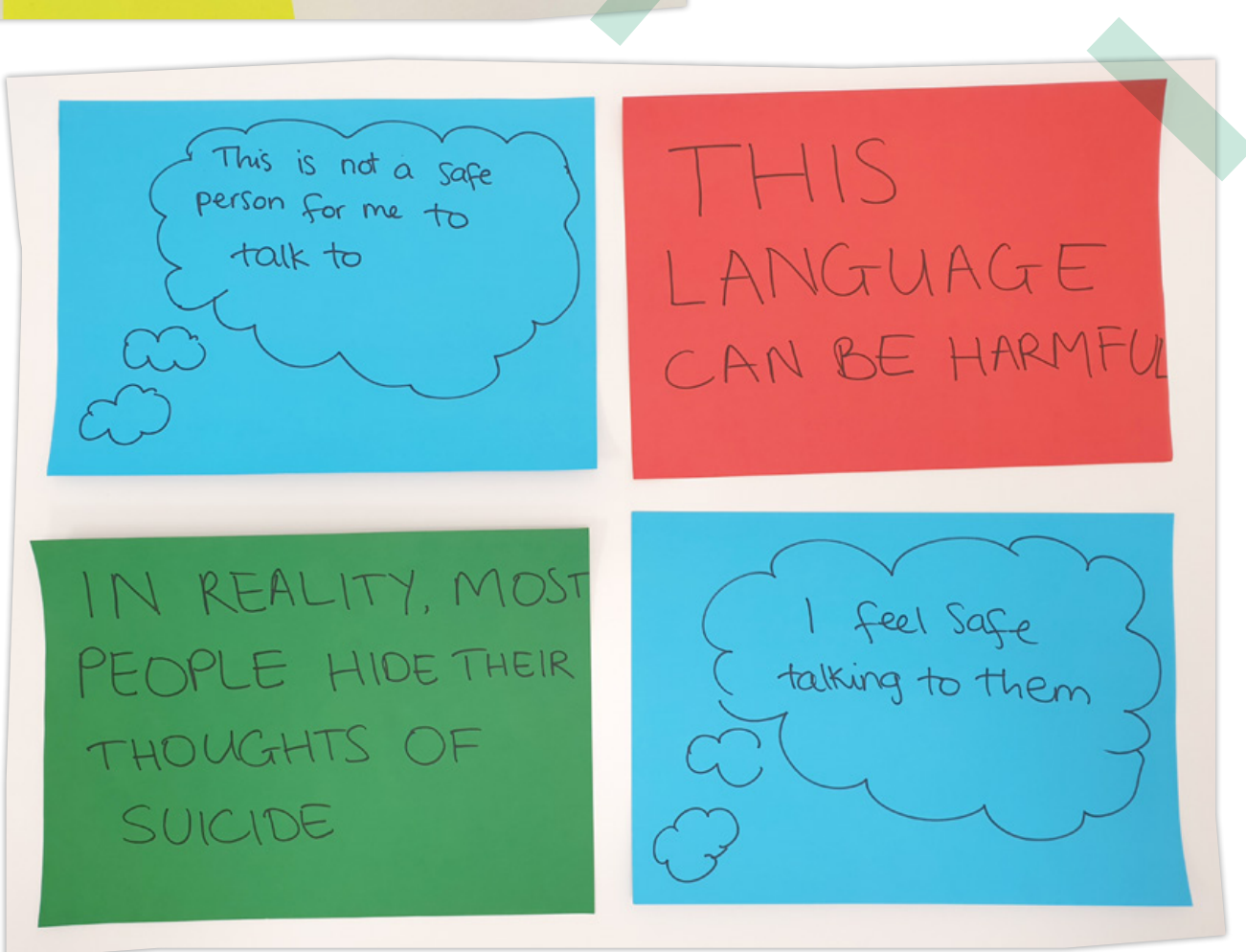

3. Reflect

Playback

- Each group/individual presents on what they have created (such as playing videos that were filmed, or presenting storyboards) and explains why they created it.
- Ideally facilitators will audio record this section or take notes.
- Facilitators can ask questions about the creative process, and provide reflection prompts, such as:
  - 'Why did you choose this concept?'
  - 'Who do you think is the target audience for this?'
  - 'If you had more time, is there anything you would do differently?'

Reflection

- Facilitated group discussion (approximately 10-15 minutes) to reflect on the topic of the day. This is an opportunity to dive deeper into their thoughts and ideas.
- You can also use this time to seek verbal feedback about the workshop, prompting questions such as:
  1. What did you think about today's workshop?
  2. What did you think about the design activity? What parts did you enjoy or find challenging?
- Ideally audio record this section, or take notes.
- If within the scope of your project, ask participants if they are interested in being contacted for future activities such as refining their ideas in a future workshop. Opportunities like this can alternatively be circulated after the workshop is complete.
- Inform participants about any next steps, such as evaluation surveys or feedback forms, if applicable.

Tip

If feedback forms are short, participants can complete them while they are still in the workshop. This could either be through a hard copy feedback form, or an online form through a QR code.

Cool down

- Participants stand or sit in a circle and reflect, answering prompts such as:
  1. What is something you learnt today?
  2. What is something you appreciated?
  3. What is something nice you're going to do tonight to look after yourself?
- Inform participants about any next steps, when to expect reimbursements, and how their ideas will be used.

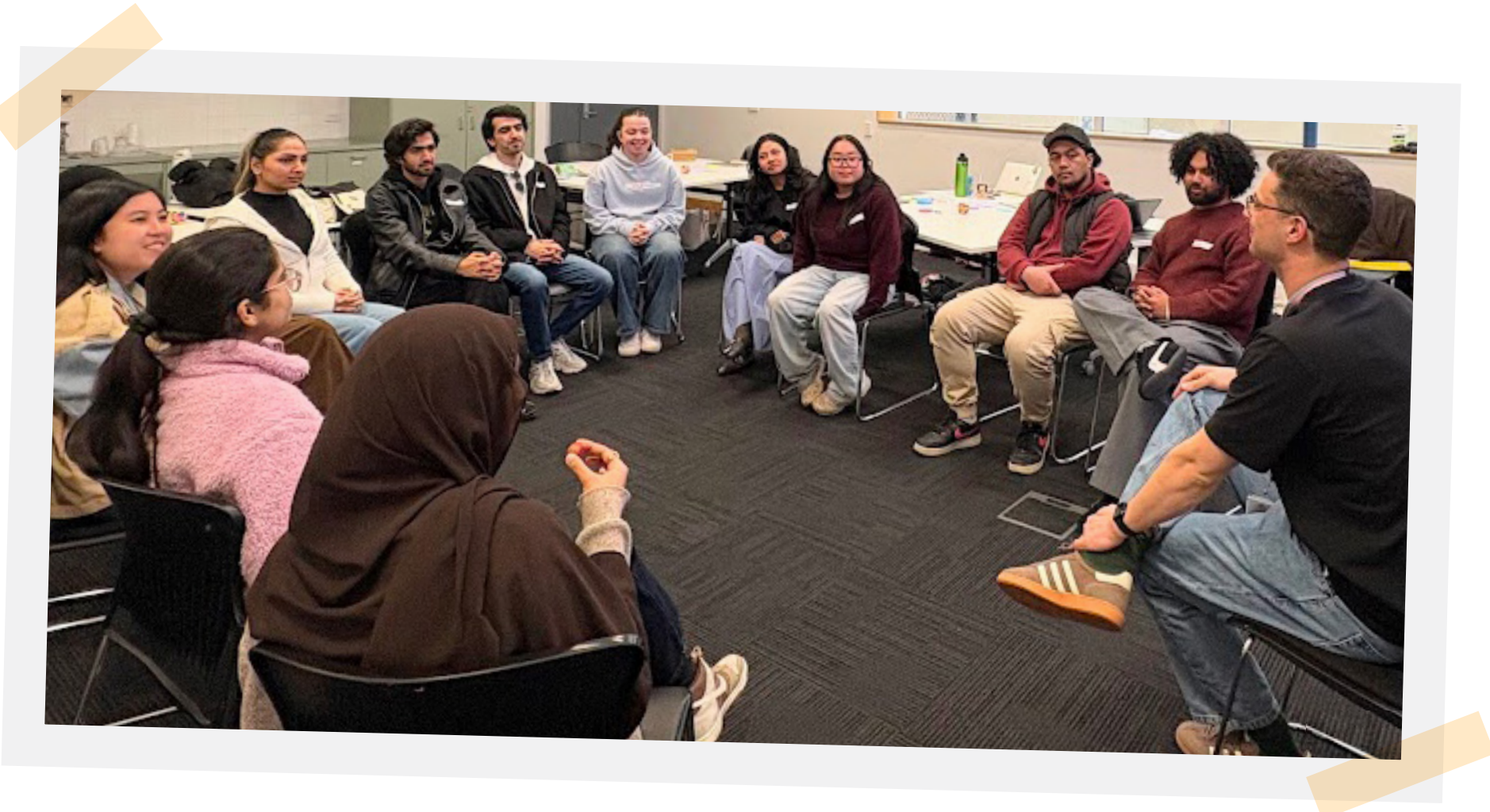

STEP 4

Reflection and feedback

Post-workshop tasks: 1-2 days after the workshop

Debriefing

Facilitators, and any other relevant project staff, should attend a debrief meeting to discuss:

- Any wellbeing concerns for the young people who attended the workshop, and if there might be any follow-up required aligned with your safety management strategy.
- Did staff feel supported during the workshop?
- What worked and what didn't work about the workshop? What can be improved in future workshops?
- Are the young people's ideas practical and achievable? Which ideas can be used as they are, and which can be adapted or iterated?
- Assigning roles for next steps:
  - post-workshop admin
  - iterating young people's ideas for Step 5 (translation)
  - organisation of any upcoming workshops.

Admin

In the days after the workshop, there are several administrative tasks.

Admin checklist:

- ✓ Email or text participants to thank them for attending the workshop, and remind them of available support numbers/services
- ✓ Organise participant payments/reimbursements
- ✓ Save/store materials created in workshop
- ✓ If applicable, send feedback survey link or any other evaluation materials
- ✓ If applicable, review feedback data and address any suggestions for improvement

STEP 5

Translating workshop ideas into actions

Now you have input from young people, you can begin incorporating their ideas into whatever you are creating. Depending on the nature of your team, this might be the responsibility of a researcher, a media and communications professional, or an external team member like a contractor.

Some ideas might be ready to go. Others might need more iterating or workshopping. For example, a participant may have suggested a concept that cannot be created within your budget, but perhaps there is a more affordable way to adapt their idea.

Even if their ideas are not feasible, sometimes you might be able to take participants' reflections and use this to inform your work.

Some ideas may not be appropriate or possible to use. This is normal.

Tip

If your budget and timeline allow, consider inviting young people back to help with this step -- like refining their ideas, creating content, or informing decisions. This is a great way to step up the participatory ladder.

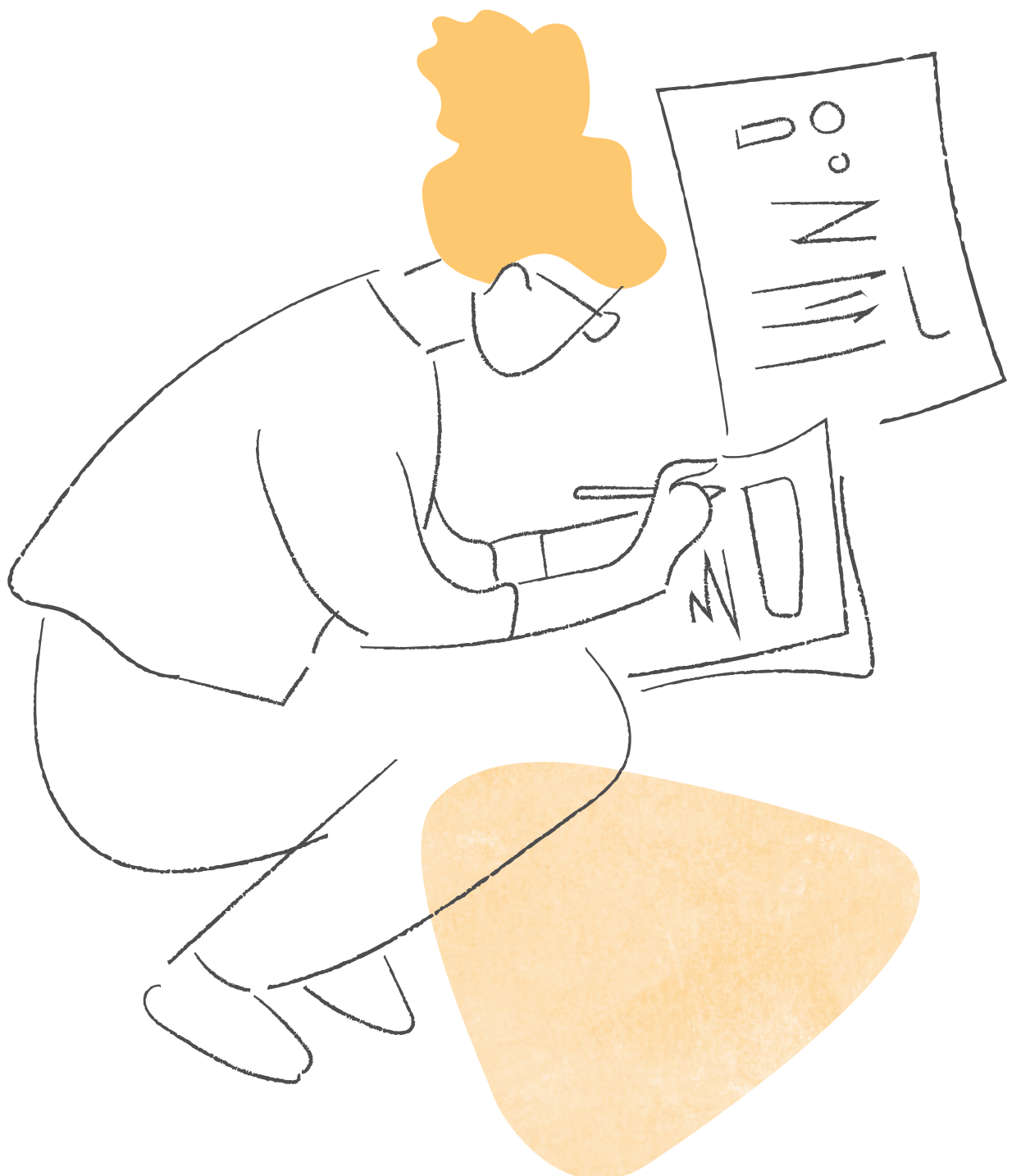

STEP 6

Closing the loop

With participants

Many participants will be interested in how their ideas were translated, and if their ideas were incorporated into the final content/product.

Consider closing the loop with participants by sending them a summary of the workshop findings and the final content/products, when it is available. This can be through an email update, plain language summary, or in other creative format e.g. a video summary featuring the facilitators.

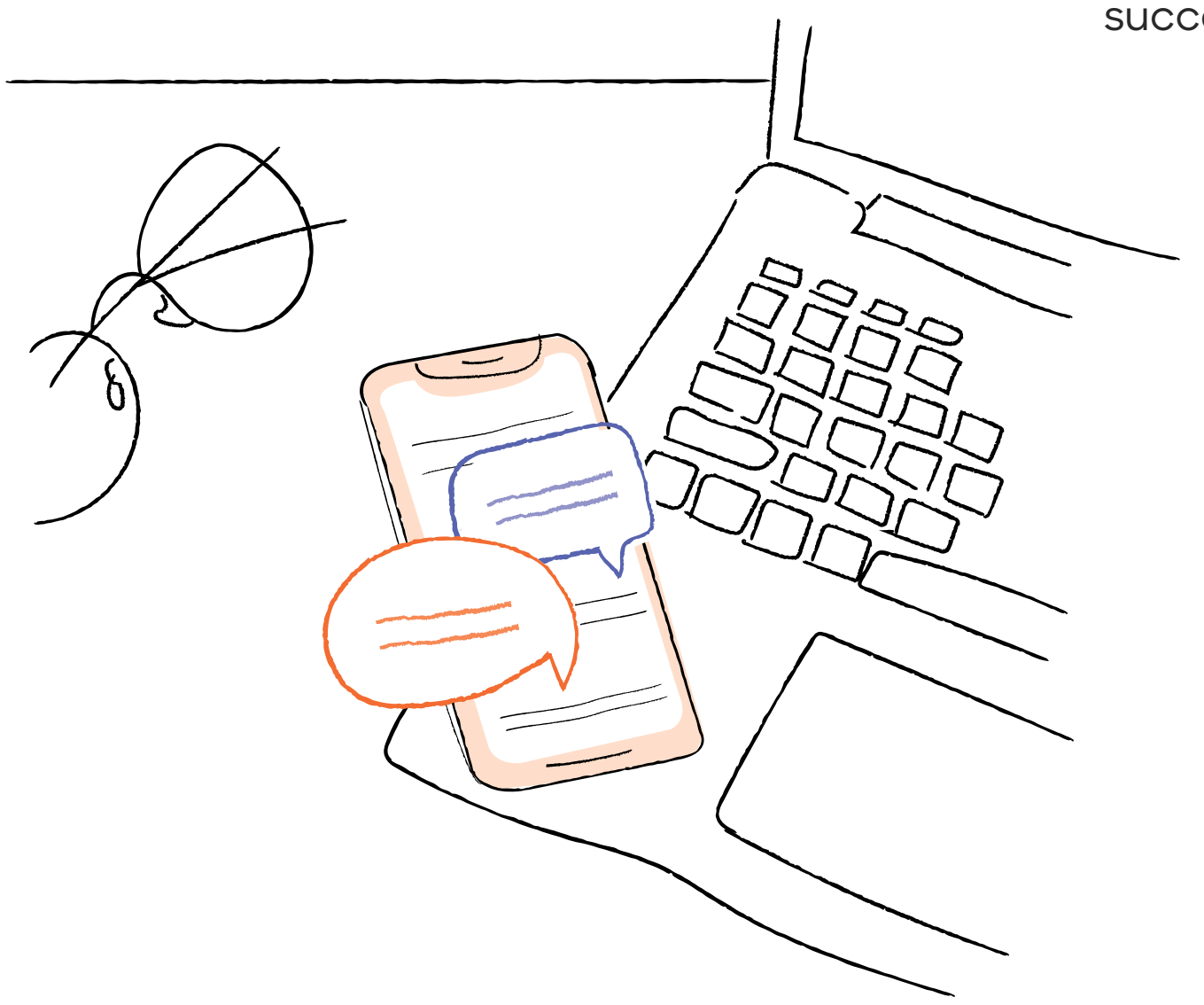

With other stakeholders

Reporting on your findings, and evaluation data, may be important to broader stakeholders. Stakeholders can include funders, team members, partner organisations and the broader community.

This ensures transparency and accountability, allowing stakeholders to see the outcomes and impacts of a project or initiative. Sharing these findings can also foster trust and confidence in the work being done.

Furthermore, it provides valuable insights and lessons learned that can inform future projects and strategies. By understanding what worked well and what didn't, you can make data-driven decisions to improve future workshops and other activities.

Lastly, reporting on any evaluation data (such as feedback from participants) can allow your team to celebrate successes and achievements.

Case study: #chatsafe social media campaign

#chatsafe is a suicide prevention initiative that seeks to help young people communicate safely online about self-harm and suicide. The #chatsafe guidelines were first created in 2018 (Robinson et al., 2018) and were accompanied by a youth suicide prevention social media campaign, created with engagement from young people.

Considering many changes in the social media landscape, the #chatsafe guidelines were updated (Robinson et al., 2023) and Edition 2 was launched in May 2023. This update called for **new social media campaigns** and **thereby new youth consultation workshops** to be conducted so that the social media campaign best reflects the latest evidence and information within Edition 2 of the #chatsafe guidelines.

It was through this project that the #chatsafe Model was refined and evaluated.

Planning and resourcing

This case study covers a series of 9 workshops completed between 2023-2025, which were attended by 107 young people. Workshop size ranged from 9-15 participants.

Workshop planning involved several months, including time required to seek ethics approval for our evaluation procedures.

Workshops were targeted towards young Australians aged 15-25 years. Some workshops were targeted towards certain population groups, including people with lived experience of suicide, LGBTIQA+ young people, people from culturally and linguistically diverse communities, and school-aged young people (aged 15-18 years).

Staff included suicide prevention researchers with experience facilitating workshops and experts in media and communication.

Several workshops were collaborative. We approached community organisations that have existing networks of young people, and invited their staff members to also attend on the day.

Participants were paid honoraria calculated at an hourly rate, aligned with our internal policies.

Recruitment

Recruitment primarily occurred through youth networks and youth-focussed organisations. We used email notices, social media advertising, and word of mouth. Participant registration was built into REDCap survey software. As part of registration, they signed a consent form (and returned a parent/guardian consent form if they were under 18), completed an image release form, and completed a short set of survey questions for use in the evaluation, relating to their self-efficacy communicating online about suicide.

Below is an example of a flyer we used to promote a workshop:

Pre-brief processes

We held pre-brief meetings in the week prior to each workshop. Our standard agenda included the following:

- Which staff are attending on the day?
- Confirm time, date, venue and any travel arrangements.
- Review registrations, including: number of registrations, demographics of participants, anything from wellness plans the team should be aware of, including lived experience of suicide and/or suicide bereavement.
- Allocating roles and responsibilities to staff members.
- What sections of the #chatsafe guidelines we want to focus on for the day.
- What design brief(s) we wish to present to participants.
- Adjusting the workshop activities based on reflections and feedback from previous workshops.

Aged 15-17 in Melbourne?
Join a #chatsafe social media co-design workshop

Share your ideas and creativity to help suicide prevention researchers design social media content to educate young people in how to have safe online conversations about self-harm and suicide.

When?
9.30am-4.30pm,
Wednesday 10 July

Where?
Orygen, 35 Poplar Rd,
Parkville VIC 3052

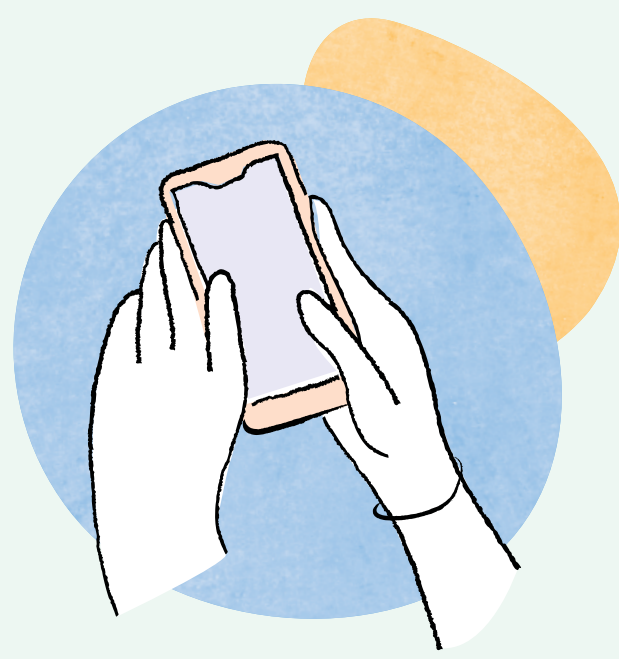

+ get reimbursed for your time

Questions? Email chatsafe@orygen.org.au

#chatsafe®

Workshop content

The workshop structure followed the general agenda on page 20.

The **Learn & Define** educational presentation included a series of slides that covered the following topics:

- Current statistics about suicide in young people
- Benefits and risks of social media for suicide prevention
- #chatsafe educational guidelines (Robinson et al., 2023) and how to talk about suicide and self-harm safely in online spaces, such as:
  - recognising harmful content
  - myth-busting
  - examples of safe language (see below).

Design activities involved:

- Brainstorming:
  - Sticky note and butchers paper activity to understand what types of content are engaging across different social media platforms, and what pages or feeds participants go to for health and mental health information.
  - Writing down a range of ideas for social media content to communicate the #chatsafe guidelines.
- Narrowing down the ideas they are most passionate about or can accomplish by the end of the day.
- Creating:
  - Storyboarding, drawing, or digitally creation (e.g. filming) of content ideas.
  - In some workshops, we highly encouraged participants to film short form content for our Instagram.

Content translation

A #chatsafe media and communications professional attended most workshops and was responsible for summarising ideas from young people, and creating #chatsafe social media content that reflected youth insights. In some instances, this involved uploading youth-created content from workshops that was ready to go. A #chatsafe team member who held professional registration as a psychologist or provisional psychologist overviewed this process, to ensure that messaging was safe and appropriate.

Some young people were invited to be an ongoing #chatsafe content creator, working with the #chatsafe team to create and refine content that was later uploaded to #chatsafe social media.

One workshop was also held with a small group of young people who attended a previous workshop, and wished to refine their initial workshop ideas. A videographer also attended this workshop to help with filming.

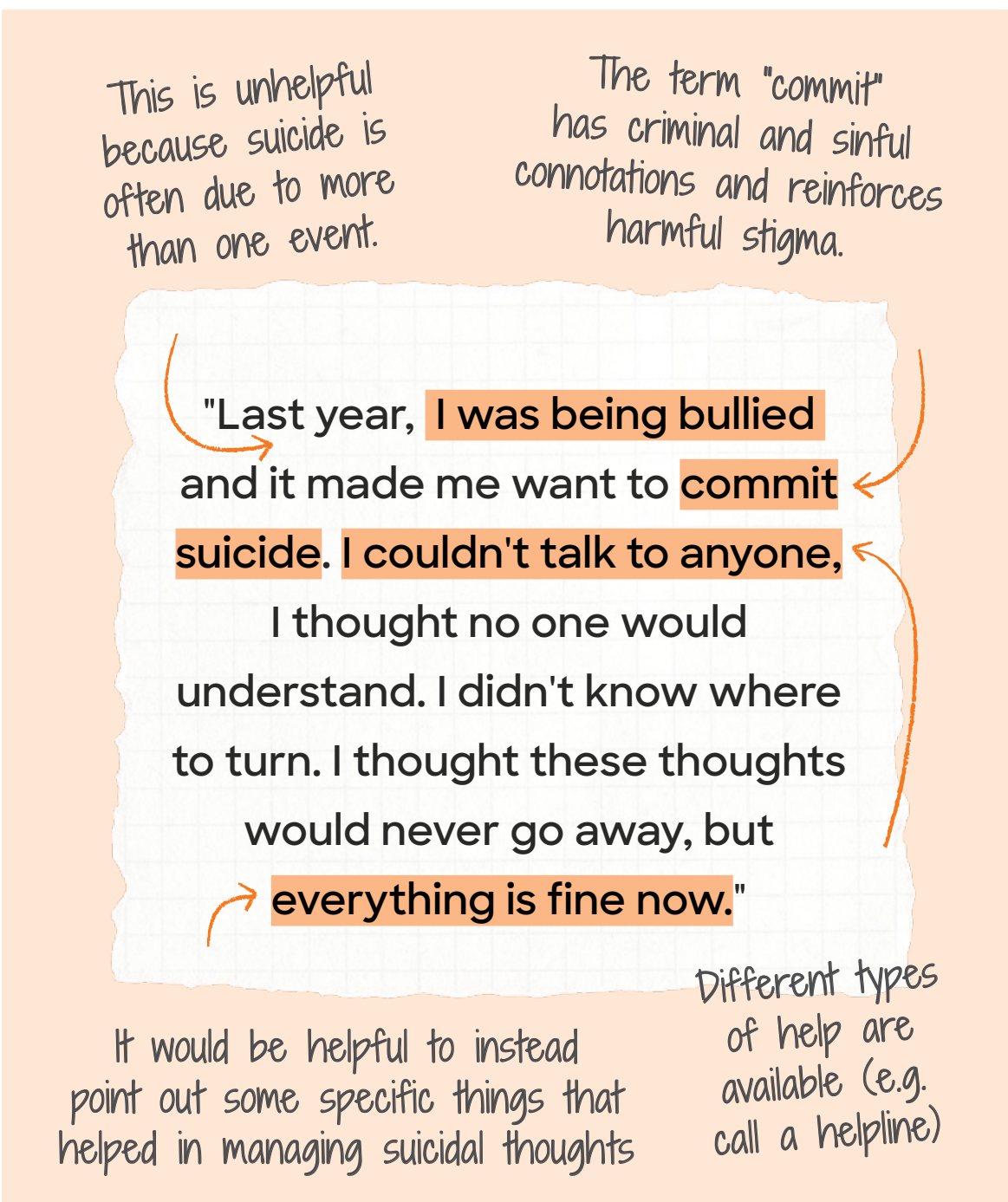

Unsafe communication example

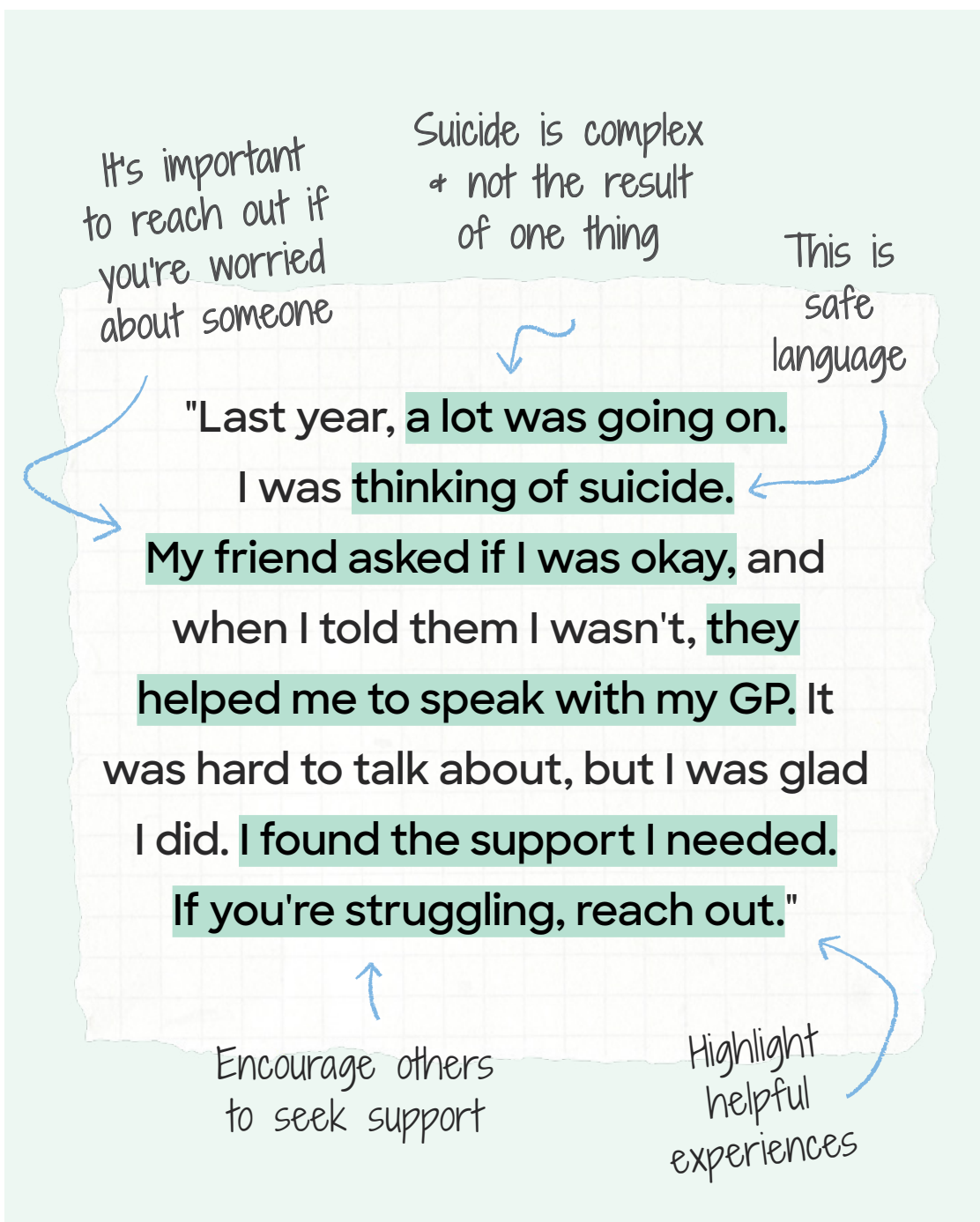

Safe communication example

Campaign achievements

The #chatsafe workshops informed two campaigns. The first #chatsafe Instagram campaign targeted young people aged 13-25, and exceeded all social media targets, reaching 960,000 young people aged 13-25 (29% of the Australian population in this age group).

A second campaign targeted school-aged young people aged 13-19, and also exceeded its targets. This campaign reached 818,000 teenagers (over half the Australian population in this age group).

Across both campaigns, youth-informed content performed particularly well, with content informed or created in our workshops among our most shared pieces of content. Pop culture references and memes helped us to get the attention of young people, and they sum-up key #chatsafe messages i.e. 'Recovery is possible', 'You should curate your social media feed so it's safe for you'.

Examples of insights and how these were translated into content:

Insight from young people | **Example content created from this insight**

You have to use memes! Something as serious as suicide needs humour to break through the tension, and to get young people's attention in crowded social media spaces

Meme from Barbie (2023) movie with relevant caption:

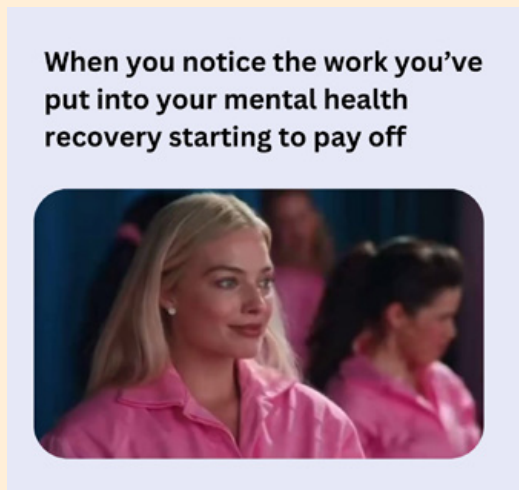

It affects me when people say KMS (kill myself) all the time

Text-based carousel posts explaining safe language:

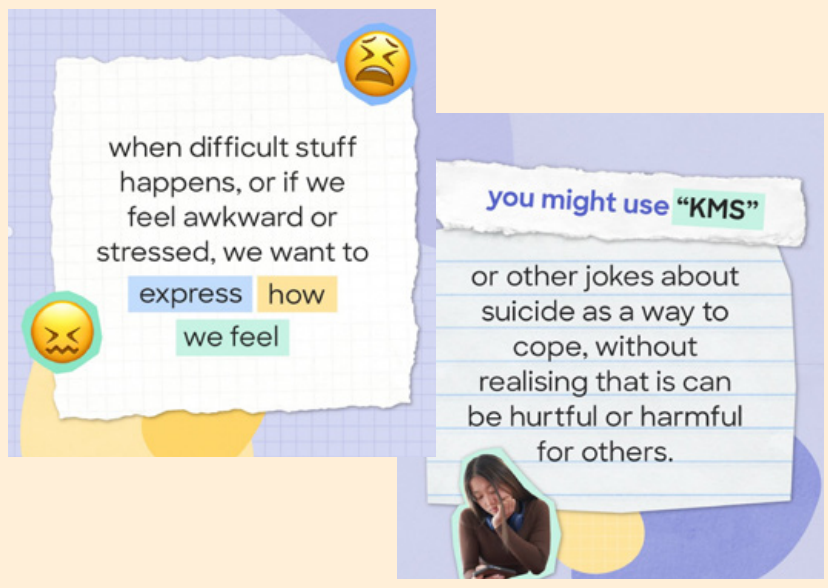

We'll take health advice from credible professionals/
The most engaging content is videos with people in them

Short form video featuring #chatsafe Research Assistant, Charlie

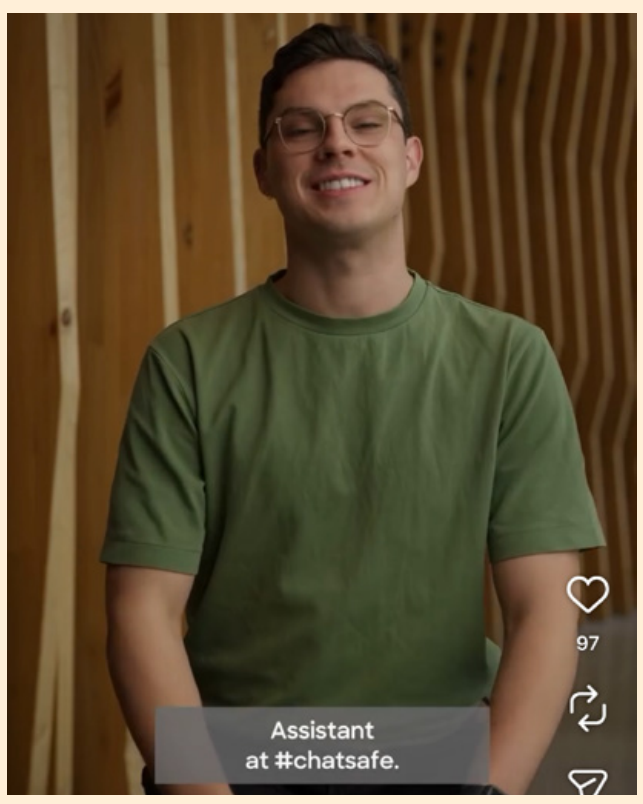

Feedback from participants

Workshop participants were invited to take part in a pre/post survey to evaluate the feasibility and acceptability of the workshops as well as their impact on self-efficacy.

All respondents who completed the post-workshop survey (87/87, 100%) agreed that they felt supported to participate in the workshop.

Most agreed that:

- their needs were met (94.3%)
- it was convenient to participate (85.1%)
- they were paid fairly (93.1%).

Workshops were **highly acceptable** to participants:

- 94.3% were satisfied or very satisfied with the workshop.
- 96.5% reported mostly enjoying the workshop, or enjoying it all.
- Suggestions to improve included allowing more time to refine ideas, and having a narrower age range per workshops. These suggestions were incorporated into latter workshops.
- There were no adverse events or safety concerns.

Plus, there were **positive impacts:**

- 78.2% agreed that the workshop led to improved knowledge about safe online communication about self-harm and suicide.
- 81.6% agreed that the workshop lead to an improvement in their ability to communicate safely online about self-harm and suicide.

Quotes from feedback surveys

> *"It was great that the researchers offered us guidance on the process but didn't dictate what we could and couldn't do. I appreciated how much they approached with curiosity over judgement and sought to understand our perspectives."*

> *"Setting up expectations at the start eased a lot of stress and allowed me to be the most creative I've ever been at a co-design workshop"*

> *"I found the environment supportive, my voice felt valued, and I felt like we did relevant work. It was also great to brainstorm with other young people."*

> *"The workshop was so accessible, the facilitators were amazing and I felt so welcomed."*

References

Aston, R., & Tse, J. (2022). *Youth Engagement Evaluation Framework*. Prepared for The Victorian Health Promotion Foundation. Centre for Program Evaluation, Melbourne Graduate School of Education, The University of Melbourne.

Robinson, J., Hill, N. T. M., Thorn, P., Battersby, R., Teh, Z., Reavley, N. J., Pirkis, J., Lamblin, M., Rice, S., & Skehan, J. (2018). The #chatsafe project. Developing guidelines to help young people communicate safely about suicide on social media: A Delphi study. PLoS ONE, 13(11). https://doi.org/10.1371/journal.pone.0206584

Robinson, J., Thorn, P., McKay, S., Hemming, L., Battersby- Coulter, R., Cooper, C., Veresova, M., Li, A., Reavley, N., Rice, S., Lamblin, M., Pirkis, J., Reidenberg, D., Harrison, V., Skehan, J., & Sala, L. La. (2023). #chatsafe 2.0. updated guidelines to support young people to communicate safely online about self-harm and suicide: A Delphi expert consensus study. PLoS ONE, 18(8 August). https://doi.org/10.1371/journal.pone.0289494

Webb, M., Cooper, C., Hemming, L., Dalton, A., Unity, E., Simmons, M., Bendall, S., & Robinson, J. (2023). Guidelines for involving young people with lived and living experience of suicide in suicide research. Orygen. https://www.orygen.org.au/Training/Resources/Self-harm-and-suicide-prevention/Guidelines/Suicide-research

Resources

Evaluation of youth participation

Aston, R., & Tse, J. (2022). Youth Engagement Evaluation Framework: Prepared for The Victorian Health Promotion Foundation. Centre for Program Evaluation, Melbourne Graduate School of Education, The University of Melbourne.

Youth participation and co-design

McKercher, K. A. (2020). Beyond sticky notes. Doing co-design for Real: Mindsets, Methods, and Movements, 1st Edn.

Orygen (n.d). Co-designing with young people: The fundamentals. https://www.orygen.org.au/Training/Resources/Service-knowledge-and-development/Guidelines/Co-designing-with-young-people-The-fundamentals/Orygen-Co-designing-with-YP-the-fundamentals?ext=.

Youth involvement in suicide prevention research

Webb, M., Cooper, C., Hemming, L., Dalton, A., Unity, E., Simmons, M., Bendall, S., & Robinson, J. (2023). Guidelines for involving young people with lived and living experience of suicide in suicide research. Orygen. https://www.orygen.org.au/Training/Resources/Self-harm-and-suicide-prevention/Guidelines/Suicide-research

Appendix

Wellness plan template

Emergency contact
(this should be a parent, guardian or another responsible adult)

Name

Relationship to you

Mobile number

Professional support person
e.g. doctor, psychologist

Name

Relationship to you

Mobile number

Any physical or medical conditions or restrictions? e.g. food allergies, asthma, epilepsy etc.

Any mental health information we may need to be aware of?

Some signs or symptoms that you notice when you are becoming uncomfortable or distressed:

Any situations activities or topics that might make you feel uncomfortable, distressed or might trigger these symptoms?

What sort of activities, tools, or resources you use to help you relax? (e.g. spending time with friends, listening to music, yoga etc):

Things you have done in the past that have helped when you were feeling unwell
e.g talking to a friend or family member, using a helpline o using strategies suggested by your psychologist.

www.orygen.org.au/chatsafe

CSV000602-ORYGEN26
