# Supplementary material for: The #chatsafe Youth Participation Model: Evaluating Participatory Workshops Involving Young People in the Design of Suicide Prevention Initiatives
Source: Health Expect. 2026 Oct 3;29(5):e70885. doi: 10.1111/hex.70885 (PMC13633697; doi:10.1111/hex.70885)
Supplement: Supplementary file 2 [file HEX-29-e70885-s001.docx]

**Additional Results**

Table S1.

Responses to feasibility feedback items at Time 2 (*n* = 87)

|  | **Item %** | | | | | **Overall %** |
| --- | --- | --- | --- | --- | --- | --- |
| **Feasibility questions** | **1 - Strongly disagree** | **Disagree** | **Neither** | **Agree** | **5 -Strongly agree** | **Agreement** |
| It was easy to participate in this workshop | 0.0 | 0.0 | 4.6 | 36.8 | 58.6 | 95.4 |
| I felt supported to participate in this workshop | 0.0 | 0.0 | 0.0 | 31.0 | 69.0 | 100.0 |
| It was convenient to participate in this workshop | 0.0 | 2.3 | 12.6 | 41.4 | 43.7 | 85.1 |
| I was fairly paid for my time to participate in this workshop | 0.0 | 0.0 | 6.9 | 32.2 | 60.9 | 93.1 |
| I felt included in the co-design process | 0.0 | 0.0 | 4.6 | 36.8 | 58.6 | 95.4 |
| My needs were met in the co-design process | 0.0 | 0.0 | 5.7 | 39.1 | 55.2 | 94.3 |
| Participating in the co-design workshop was useful | 0.0 | 1.1 | 8.0 | 37.9 | 52.9 | 90.8 |
| Participating in co-design workshop was worth the time and effort | 0.0 | 0.0 | 3.4 | 36.8 | 59.8 | 96.6 |
| I felt I connected socially and meaningfully with other young people in this workshop | 0.0 | 3.4 | 12.6 | 32.2 | 51.7 | 83.9 |
| A diverse range of voices and experiences were included in these workshops | 0.0 | 1.1 | 12.6 | 29.9 | 56.3 | 86.2 |
| These workshops were representative of my opinions | 0.0 | 0.0 | 11.5 | 39.1 | 49.4 | 88.5 |

Table S2.

Responses to acceptability feedback items at Time 2 (*n* = 87)

|  | **Item** | | | | | **Overall** |
| --- | --- | --- | --- | --- | --- | --- |
| **Acceptability question (‘Please rate the degree to which you were satisfied/enjoyed with the following aspects of the co-design workshop’)** | **1 – Not satisfied/ did not enjoy** | **Disagree** | **Neither** | **Agree** | **5 -Very satisfied / enjoyed** | **% satisfied / enjoyed** |
| Facilitators | 0.0 | 1.1 | 1.1 | 20.7 | 77.0 | 97.7 |
| Design activities | 1.1 | 0.0 | 3.4 | 42.5 | 52.9 | 95.4 |
| Connecting socially with other young people | 1.1 | 0.0 | 11.5 | 37.9 | 49.4 | 87.3 |
| Working collaboratively with other young people | 0.0 | 1.1 | 8.0 | 28.7 | 62.1 | 90.8 |
| Connecting with professionals in the field | 0.0 | 1.1 | 12.6 | 37.9 | 48.3 | 86.2 |
| Creating social media content | 0.0 | 0.0 | 12.6 | 29.9 | 57.5 | 87.4 |
| Learning about #chatsafe | 0.0 | 0.0 | 3.4 | 40.2 | 56.3 | 96.5 |
| Resources available | 0.0 | 0.0 | 9.2 | 37.9 | 52.9 | 90.8 |

Table S3.

Responses to perceived safety items at Time 2 (*n* = 87)

|  | **Item** | | | | | **Overall** |
| --- | --- | --- | --- | --- | --- | --- |
| **Perceived safety question** | **1 - Strongly disagree** | **Disagree** | **Neither** | **Agree** | **5 -Strongly agree** | **% Agreement** |
| The #chatsafe co-design workshop made me feel upset. | 63.2 | 20.7 | 10.3 | 3.4 | 2.3 | 5.7 |
| The #chatsafe co-design workshop made me feel suicidal. | 90.8 | 5.7 | 1.1 | 1.1 | 1.1 | 2.2 |
| The #chatsafe co-design workshop made me want to self-harm. | 89.7 | 8.0 | 0.0 | 1.1 | 1.1 | 2.2 |
| I felt emotionally/ psychologically safe taking part in the #chatsafe co-design workshop | 1.1 | 0.0 | 2.3 | 40.2 | 56.3 | 96.5 |
| I felt culturally safe taking part in the #chatsafe co-design workshop | 2.3 | 0.0 | 4.6 | 32.2 | 60.9 | 93.1 |
