# Supplementary material for: The #chatsafe Youth Participation Model: Evaluating Participatory Workshops Involving Young People in the Design of Suicide Prevention Initiatives
Source: Health Expect. 2026 Oct 3;29(5):e70885. doi: 10.1111/hex.70885 (PMC13633697; doi:10.1111/hex.70885)
Supplement: Supplementary file 3 [file HEX-29-e70885-s002.docx]

Table 1. Evaluation domains of interest and measurements

| **Research Question** | **Outcome of Interest** | **Domain** | **Operationalisation** | **Measurement (timepoints)** |
| --- | --- | --- | --- | --- |
| Is it feasible to conduct #chatsafe workshops with a broad range of young people? | Feasibility | Reach | Number of young people reached through the sampling approach and invited to participate in the workshop | Registration data: Number of young people expressing interest in the workshop (completing Time 1 [T1])   Number of organisations who express interest and participated  Demographic profile of young people/subgroups of young people who expressed interest in participating. |
|  |  | Participation/Uptake | Number of young people who participate in the workshop | Number of young people who attended a workshop  Demographic profile of young people/subgroups of young people who participated, including for priority population workshops (T1) |
|  |  | Support/Reactions | Participants’ perceptions of accessibility of the workshops. | Self-reported perceived support and accessibility in the workshop (Time 2 [T2])  Self-reported convenience of participation, as well as feelings towards remuneration (T2) |
| Do young people find it acceptable to participate in #chatsafe workshops? | Acceptability | Appropriateness | Participants’ perceptions of workshops’ relevance and inclusivity, as well as feelings of needs being met | Self-reported feelings of inclusion (T2)  Self-reported feelings of needs being met (T2) |
|  |  | Attitudes | Participants’ reporting of attitudes and feelings toward the participatory process | Self-reported feelings of positive attitudes and enjoyment (T2) |
|  |  | Burden | Participants reporting of workshops being worth the time and effort | Self-reported feelings of workshops being worthwhile and a good use of time (T2) |
|  |  | Perceived effectiveness | Participants perceptions of workshops’ usefulness, helpfulness and whether it may achieve a purpose | Self-reported feelings of the workshops being useful and achieving their purpose (T2) |
| Is participating in a #chatsafe workshop safe for young people? | Safety | Distress | Participants self-reported feelings of distress, and self-harm/suicidal ideation as a result of participating | Self-reported feelings of distress from young people (T2) |
|  |  | Adverse events | Any adverse events (e.g. an escalation in suicide risk) associated with participating in a workshop | Number of adverse events occurring during workshops |
| Does participation in a #chatsafe workshop increase young people’s perceived self-efficacy when communicating online about self-harm or suicide? | Self-efficacy | Confidence | Participants’ confidence levels in communicating safely online regarding self-harm and suicide | Change in self-reported confidence in communicating online about self-harm or suicide (T1+T2) |
|  |  | Perceived knowledge | Participants’ perceived learnings as a result of participating in a workshop | Self-reported perceived changes in knowledge regarding safe online communication about self-harm and suicide as a result of participating in a workshop (T2) |
|  |  | Perceived skill | Participants’ perceived changes in their ability to communicate safely online about self-harm and suicide | Self-reported changes in perceived ability to communicate safely online about self-harm or suicide (T2) |
